# Climate Change Impacts on the Distribution Patterns and Habitat Suitability of the Oriental Stork ( *Ciconia boyciana* ) in China: Implications for the “30 × 30” Conservation Targets

**DOI:** 10.1002/ece3.74424

**Published:** 2026-09-28

**Authors:** Pengzhou Chen, Haoyu Ma, Yue Fang, Jiaxiang Chang, Donglai Hua, Hao Liu

**Affiliations:** ^1^ School of Life Sciences (School of Ecological Forestry) Mianyang Teachers' College Mianyang China; ^2^ Sichuan Jiuzhai Valley National Ecological Quality Comprehensive Monitoring Station Mianyang China; ^3^ Forest Ecology and Conservation in the Upper Reaches of the Yangtze River Key Laboratory of Sichuan Province Mianyang China; ^4^ Key Laboratory of Research and Conservation of Biological Diversity in Minshan Mountain of National Park of Giant Pandas at Mianyang Teachers' College of Sichuan Provincial Department of Education Mianyang China; ^5^ Ecological Security and Protection Key Laboratory of Sichuan Province Mianyang Normal University Mianyang China

**Keywords:** climate change, HSI‐InVEST, MaxEnt, Oriental Stork, priority conservation areas, suitable distribution area

## Abstract

The Oriental Stork ( *Ciconia boyciana* ), a wetland flagship species, faces threats from climate change and human activities. Assessing habitat suitability dynamics and identifying priority conservation areas are essential for implementing the “30 × 30” target of the Kunming‐Montreal Global Biodiversity Framework. We integrated an optimized MaxEnt model with the HSI‐InVEST habitat quality model to predict potential suitable habitats and key drivers for this species in China under current and future climate scenarios (SSP1‐2.6, SSP2‐4.5, SSP5‐8.5, 2041–2100), and delineated priority conservation areas. The optimized MaxEnt model performed excellently (mean AUC = 0.943). Six dominant factors were identified: altitude (suitable range: −3.5 to 11.7 m), precipitation of the wettest month, distance to lakes, distance to roads, mean diurnal temperature range, and precipitation of the coldest quarter. Currently, suitable habitat covers 106.57 × 10^4^ km^2^ (11.1% of China's land area), mainly in eastern China. Under future scenarios, suitable habitat is projected to expand (0.22%–7.82%) and the centroid of potentially suitable habitat is projected to shift northeastward (centroid migration: 16.82–77.22 km), with precipitation as a key driver. Overlaying MaxEnt predictions with HSI‐InVEST habitat quality identified priority areas (suitability ≥ 0.4, habitat quality index ≥ 0.8), concentrated in lake beach and floodplain wetlands of the North China Plain, Huang‐Huai‐Hai Plain, and Jianghuai region. We recommend designating these priority areas as core conservation zones with strict spatial control and hydrological regulation, and planning ecological corridors in northeastern expansion zones. This multi‐model coupling framework provides theoretical support for optimizing wetland waterbird conservation strategies.

## Graphical abstract

Using an optimized MaxEnt model integrated with the HSI‐InVEST habitat quality model, we predicted that suitable habitats for the Oriental Stork in China will expand (0.22%–7.82%) and shift northeastward under future climate scenarios, with altitude and precipitation identified as key drivers. Priority conservation areas (suitability ≥ 0.4, habitat quality ≥ 0.8) are concentrated in lake beach and floodplain wetlands of the North China Plain, Huang‐Huai‐Hai Plain, and Jianghuai region. We recommend designating these areas as core conservation zones with strict spatial control and hydrological regulation, while planning ecological corridors in northeastern expansion zones to support the “30 × 30” conservation targets.

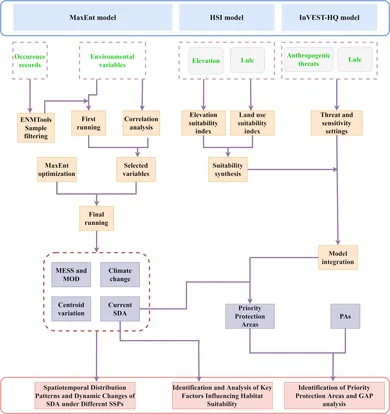

## 1 Introduction

Wetland birds are a key indicator group for the health of global ecosystems, playing an irreplaceable role in maintaining biodiversity and ecological balance (Green and Elmberg 2013). The Sixth Assessment Report of the Intergovernmental Panel on Climate Change (IPCC) explicitly states that climate change is a major threat to global biodiversity (Jonathan and Nina 2021). Due to an increased understanding of the ecosystem services provided by wetlands and the growing recognition of their value as flagship conservation species, global attention toward rare waterbirds such as the Oriental Stork is increasing, and conservation efforts are being continuously strengthened. As a flagship species of East Asian wetland ecosystems, the Oriental Stork population faces persistent threats from habitat loss and fragmentation, agricultural development, climate change, and human disturbance. For such rare and endangered species, the most effective conservation strategy involves securing their living space through the establishment of nature reserves, restoration of key habitats, and planning of ecological corridors to reverse the declining trend of its population. The Kunming‐Montreal Global Biodiversity Framework explicitly proposes the “30 × 30” target (CBD 2022), aiming to protect at least 30% of the world's terrestrial and marine areas by 2030. This further underscores the urgency and strategic importance of accurately identifying priority conservation areas and optimizing the spatial layout of protected areas.

The Oriental Stork is a large wading bird endemic to East Asia. It is currently classified as Endangered (EN) on the IUCN Red List, with a global population of approximately 1000–2499 mature individuals. The species breeds in the Amur and Ussuri basins along the border of Russia and northeast China, while its main wintering grounds are in the lower Yangtze basin and southern China (Zheng et al. 2016). It is a fully migratory species. In terms of habitat preference, the Oriental Stork forages in open, shallow freshwater wetlands and rice paddies, and nests on tall trees or artificial structures. The species faces multiple persistent threats, including wetland drainage, overfishing, and human disturbance. Climate change further exacerbates these pressures: studies have shown that suitable habitat for the species could decline significantly under future scenarios, and drought events have been found to limit population size in critical wintering sites such as Poyang Lake (Zhou et al. 2025).

Given the multiple threats facing the Oriental Stork and the broader context of ongoing global habitat loss, the quality of critical habitats for many species is declining rapidly, leading to a crisis in species diversity conservation (Zhao et al. 2024). Therefore, timely assessment of habitat suitability is of great significance for protecting biodiversity (Carter et al. 2005). In the context of climate change, clarifying changes in the potential distribution patterns of species is crucial for measuring the impacts of climate change on species and formulating conservation strategies to maintain ecological balance (Deb et al. 2017). In recent decades, Species Distribution Models (SDMs) have been widely used to predict suitable habitats for species (Duarte et al. 2019; Elith and Franklin 2019). Among these, the MaxEnt model has attracted considerable attention due to its powerful predictive ability and high spatial consistency (Phillips et al. 2006). Compared with other models, the MaxEnt model requires a relatively low amount of species occurrence data, is cost‐effective, easy to operate, has fast computational speed, and generally achieves better predictive performance (Costa et al. 2010). Previous studies have shown that the maximum entropy model can effectively assess the extent of suitable habitat for the Oriental Stork (Li et al. 2023).

In the context of ongoing global habitat loss (IPCC 2014), identifying priority areas for conservation and development remains a key strategy for optimizing nature reserve management and achieving biodiversity targets (Ma et al. 2021). The habitat quality module of the InVEST model, which requires relatively little data and offers high assessment accuracy, has become a commonly used tool for habitat quality evaluation. This model can quantify current habitat quality and the degradation risks arising from land‐use change and human disturbance. When integrated with land‐use analysis, it can effectively delineate priority conservation areas. However, relevant studies have often focused on the spatiotemporal evolution characteristics of habitat quality and its interactions with urbanization, land use, and landscape patterns (Bai et al. 2020; Chang et al. 2021), while research on the variation of habitat quality along topographic gradients remains insufficient (Lei et al. 2022). As an important natural geographical element, topography is one of the core driving factors influencing the spatial differentiation of regional habitat quality (Xu et al. 2020). The Habitat Suitability Index (HSI) model is a widely applied method for assessing habitat quality and evaluating potential development impacts (Hightower et al. 2012). First proposed by the National Wetlands Research Center and the Fish and Wildlife Service of the U.S. Geological Survey in the 1980s (Yu et al. 2015), the HSI model is valued for its simplicity, ease of interpretation, and robust performance. It can predict the probability of species occurrence based on environmental variables affecting species distribution (Hirzel and Lay 2008; Elith et al. 2011), and its outputs can be integrated with Geographic Information Systems (GIS) to provide practical decision‐support tools for managers (Vinagre et al. 2006). For assessing the habitat quality of the Oriental Stork, HSI modeling can effectively compensate for the InVEST model's inability to fully account for topographic factors. The integration of these two models is expected to achieve a more comprehensive habitat assessment.

In this context, the present study aims to achieve three main objectives: (1) to analyze the extent of suitable distribution areas for the Oriental Stork under different climate scenarios and assess their spatiotemporal dynamics; (2) to identify the key factors influencing the distribution of this species; and (3) to integrate the predictions of the MaxEnt model with the HSI‐InVEST integrated model to identify priority conservation areas for the species. The research findings will deepen the understanding of how the Oriental Stork responds to climate change and other environmental pressures, providing a fundamental baseline reference for biodiversity conservation, species habitat management, and the optimization of wetland waterbird conservation strategies under the targets of the Kunming‐Montreal Global Biodiversity Framework.

## 2 Materials and Methods

### 2.1 Research Framework

In this study, an integrated modeling workflow was constructed by combining the MaxEnt model with the HSI‐InVEST integrated model. This framework can clearly map the potential distribution range of the Oriental Stork under current bioclimatic conditions and future scenarios, and identify priority conservation areas for this species (Figure 1).

**FIGURE 1:**
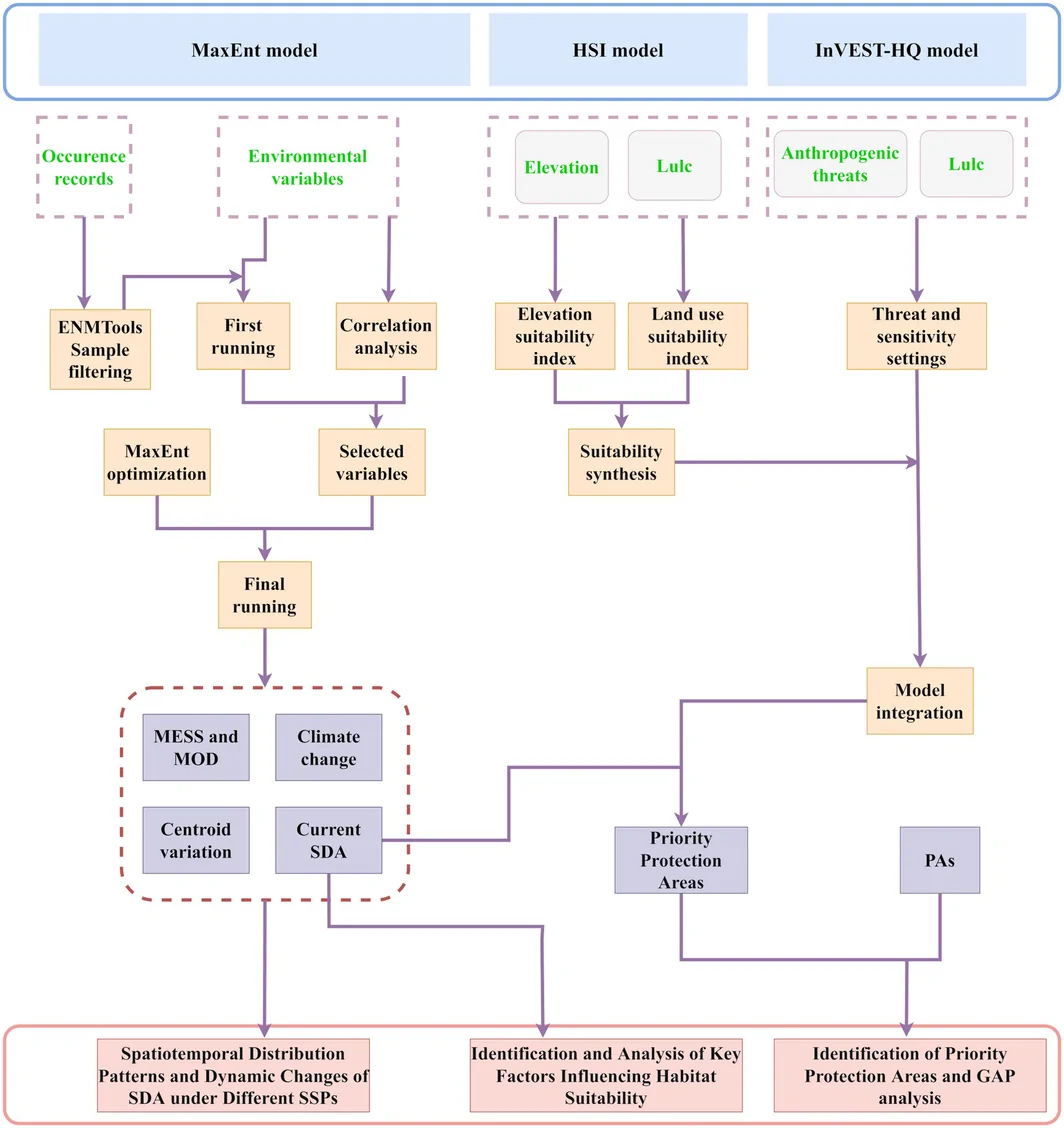
Study flow.

### 2.2 Study Area

China (Figure 2) is located in eastern Asia, spanning 3°51′–53°33′ N and 73°33′–135°05′ E, extending approximately 5200 km east–west and 5500 km north–south, with a terrestrial area of about 9.6 million km^2^—the fourth‐largest country in the world. Mainland China is bordered to the east and south by the Bohai Sea, Yellow Sea, East China Sea, and South China Sea, with a coastline of 18,000 km, and shares a 22,000 km land border with 14 countries. The country's topography features a distinct staircase‐like descent from west to east, comprising high mountains, plateaus, basins, plains, and hills. This diverse geographical structure, combined with a wide latitudinal range spanning cold‐temperate to tropical zones, dense river and lake systems, and the coexistence of forests, meadows, and wetlands, creates ideal habitats for rare bird species such as the Oriental Stork.

**FIGURE 2:**
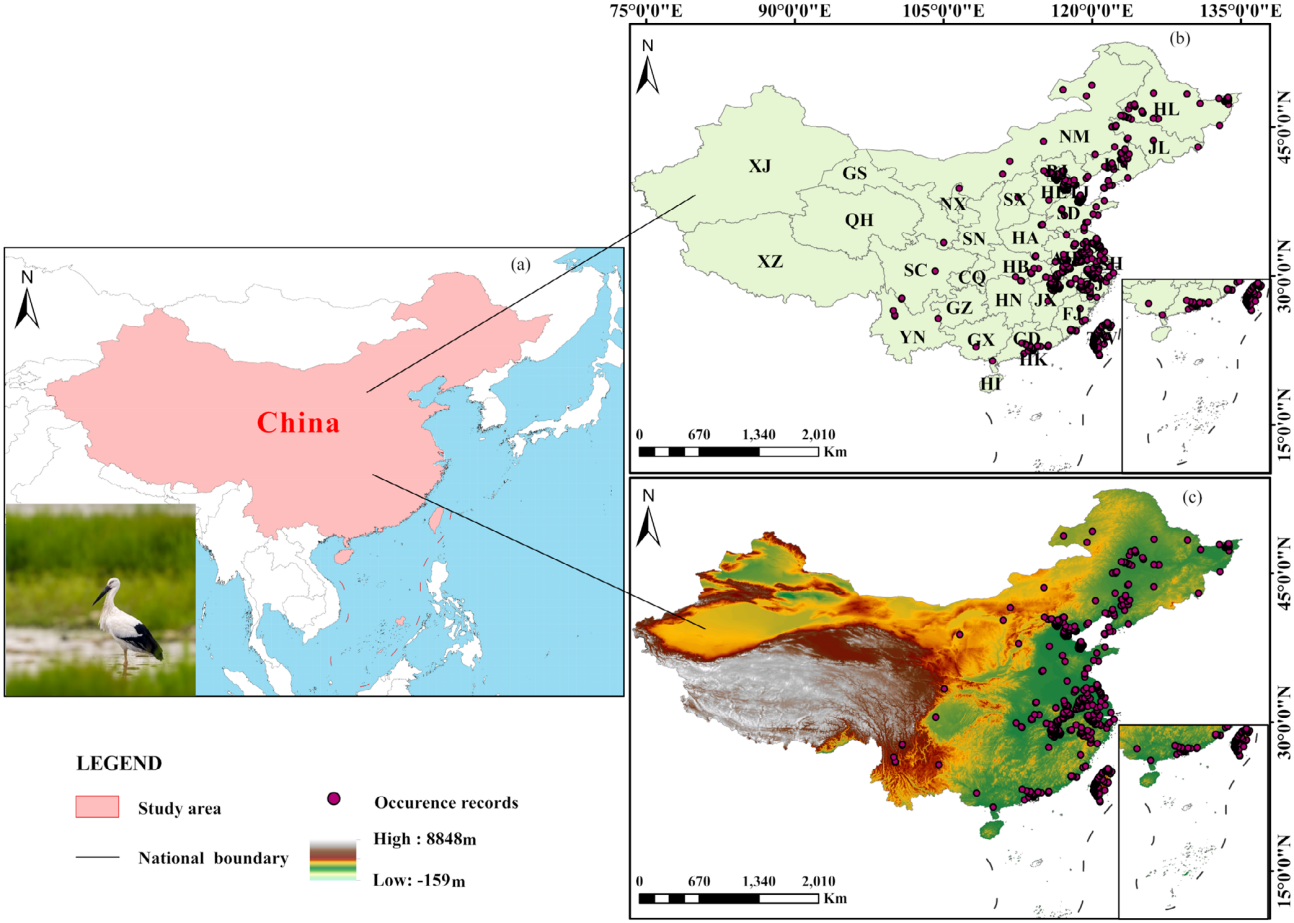
Study area. (a) location of China. (b) provincial administrative divisions and the occurrence records of *Ciconia boyciana* . (c) occurrence records of *Ciconia boyciana* on a digital elevation model (DEM). China Administrative Map from Resource and Environmental Science Data Platform (https://www.resdc.cn/). GS (2024)0650. The Oriental Stork photograph in panel (a) was taken by Yan Zhiqiang in Sichuan Province on April 3, 2026. The Chinese Provinces are abbreviated as follows: YN, Yunnan; GX, Guangxi; GD, Guangdong; HI, Hainan; TW, Taiwan; FJ, Fujian; ZJ, Zhejiang; JX, Jiangxi; JS, Jiangsu; AH, Anhui; HN, Hunan; HB, Hubei; HA, Henan; HE, Hebei; SX, Shanxi; SD, Shandong; GZ, Guizhou; CQ, Chongqing; SC, Sichuan; XZ, Xizang; XJ, Xinjiang; QH, Qinghai; GS, Gansu; NX, Ningxia; SN, Shaanxi; NM, Neimenggu; LN, Liaoning; JL, Jilin; HL, Heilongjiang.

### 2.3 Model Data Preprocessing and Construction

#### 2.3.1 Species Distribution Data

Occurrence data for the Oriental Stork were compiled from the Global Biodiversity Information Facility (GBIF, https://www.gbif.org/) and the China Bird Watching Record Center (http://www.birdreport.cn/), yielding an initial total of 3421 records. Raw sampling points are often concentrated in specific geographical areas, and directly using densely distributed points can lead to spatial autocorrelation, causing overfitting in MaxEnt model results (Ma et al. 2024). To reduce bias caused by clustering effects, we used ENMTools 1.0 (Warren et al. 2010) to screen the data, applying the grid‐cell based thinning method with a grid resolution of 2.5 arc‐minutes (matching the resolution of the environmental raster layers), and retaining only the most representative record within each grid cell; additionally, duplicate records with identical longitude and latitude were removed (Deb et al. 2017), and anomalous points falling outside the known geographical distribution range of the species were excluded, thereby eliminating spatial bias from repeated reporting or oversampling. After this spatial filtering process, 668 occurrence points were retained for subsequent modeling (Huang et al. 2022). The filtered data were organized into CSV format with three columns (species name, longitude, latitude) to meet the input requirements of MaxEnt.

#### 2.3.2 Acquisition and Processing of Environmental Factors

##### 2.3.2.1 Data for the MaxEnt Model

We considered the impacts of climate, topography, hydrology, human disturbance, and land cover on the habitat suitability of the Oriental Stork, and selected an initial set of 27 environmental factors following established norms in waterbird habitat research (Sun et al. 2021; Wang et al. 2023) (Table 1). All raster data were preprocessed in ArcGIS 10.8: uniformly projected to WGS_1984, resampled to 2.5 arc‐minute resolution, and clipped to China's administrative boundary, then converted to ASCII format for MaxEnt input. Future climate data (2041–2100) were derived from the BCC‐CSM2‐MR model within CMIP6 under three SSP scenarios (SSP1‐2.6, SSP2‐4.5, SSP5‐8.5), each including three periods (2041–2060, 2061–2080, 2081–2100), and were maintained at the same resolution and coordinate system as current data to ensure comparability. Importantly, all non‐climatic environmental variables—including topography, hydrology, land use, roads, nighttime lights, and vegetation cover—were held constant under future scenarios. This is a simplifying assumption that allows our study to specifically isolate the effects of climate change on the species' potential climatic suitability, rather than attempting to predict actual future habitat availability. We acknowledge that future land‐use changes and infrastructure development may further constrain or, in some cases, alter actual habitat availability beyond the climate‐induced shifts projected here. To mitigate overfitting risk from multicollinearity (Sillero 2011; Merow et al. 2013), a two‐step screening procedure was applied: first, variables with average contribution < 1% from the Jackknife test were excluded (Wei et al. 2019); second, Pearson correlation analysis was performed on retained variables using ENMTools (Figure 3), and for pairs with |*r*| ≥ 0.8 (De Marco and Nóbrega 2018), priority was given to retaining the variable with higher contribution rate and clearer ecological significance (Sheppard 2013). Through this screening, 10 environmental variables were ultimately selected for subsequent modeling (Table 1), preprocessed in ArcGIS, converted to ASCII format using SDMtoolbox 2.6, and imported into MaxEnt for computation.

**TABLE 1:** Environmental variables considered for MaxEnt modeling of *Ciconia boyciana* .

Variables type	Variables name	Description	Unit	MaxEnt modeling	Source and website
Climatic and topographic variables	Bio1	Annual Mean Temperature	°C		WorldClim (https://worldclim.org/)
	Bio2	Mean Diurnal Range	°C	●	
	Bio3	Isothermality	%		
	Bio4	Temperature Seasonality			
	Bio5	Max Temperature of Warmest Month	°C		
	Bio6	Min Temperature of Coldest Month	°C		
	Bio7	Temperature Annual Range	°C		
	Bio8	Mean Temperature of Wettest Quarter	°C		
	Bio9	Mean Temperature of Driest Quarter	°C		
	Bio10	Mean Temperature of Warmest Quarter	°C		
	Bio11	Mean Temperature of Coldest Quarter	°C		
	Bio12	Annual Precipitation	mm		
	Bio13	Precipitation of Wettest Month	mm	●	
	Bio14	Precipitation of Driest Month	mm		
	Bio15	Precipitation Seasonality	%	●	
	Bio16	Precipitation of Wettest Quarter	mm		
	Bio17	Precipitation of Driest Quarter	mm		
	Bio18	Precipitation of Warmest Quarter	mm		
	Bio19	Precipitation of Coldest Quarter	mm	●	
	Elevation		m	●	RESDC (https://www.resdc.cn/)
Water resource variables	Distance from river	—	m		OpenStreetMap (https://www.openstreetmap.org/)
	Distance from lake	—	m	●	
Anthropogenic disturbance variables	Distance from road	—	m	●	
	Distance from settlement	—	m		
	NTL	Nighttime Light Index		●	RESDC (https://www.resdc.cn/)
Land cover and vegetation variables	FVC	Fractional Vegetation Cover	—	●	TP‐DC (https://data.tpdc.ac.cn/)
	LULC	Land Use and Land Cover	—	●	RESDC (https://www.resdc.cn/)

*Note:* ● used for Maxent model modeling.

**FIGURE 3:**
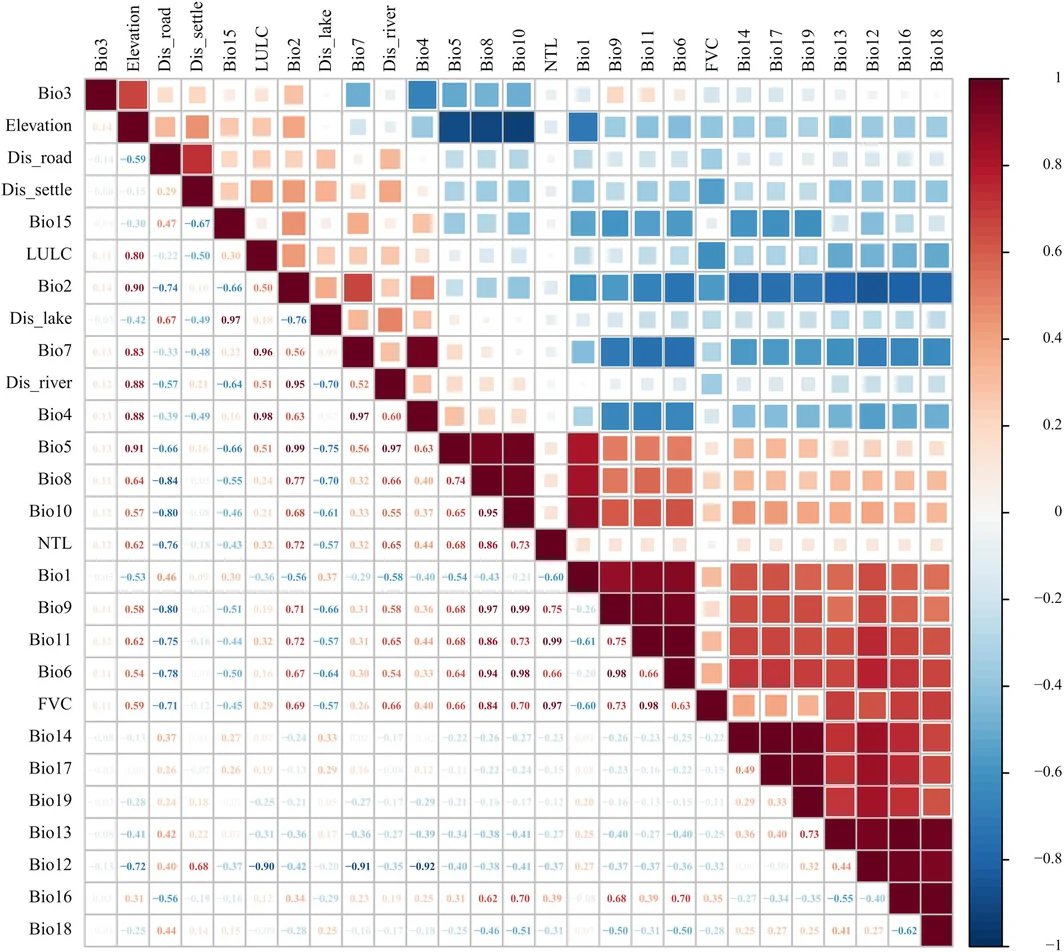
Pearson correlation analysis.

##### 2.3.2.2 Data for the HSI‐InVEST Integrated Model

Land Use and Land Cover (LULC) data were obtained from the Resource and Environment Science and Data Platform (RESDC, https://www.resdc.cn/) and reclassified into 17 land use types (Paddy field, Forest land, Grassland, River and canal, Lake, Reservoir and pond, Permanent glacier, Mudflat, Floodplain, Construction land, Sandy land, Gobi, Saline‐alkali land, Marshland, Bare land, Bare rock, and Other land) to meet the input requirements of the habitat quality assessment model. Specifically, the LULC data used in this study are derived from the China Multi‐Period Land Use Remote Sensing Monitoring Dataset (CNLUCC), produced by the Institute of Geographic Sciences and Natural Resources Research, Chinese Academy of Sciences. The dataset was constructed through manual visual interpretation based on Landsat remote sensing images, using a two‐level classification system with 6 primary types and 23 secondary types. The data used in this study correspond to the 2020 annual product at 1 km × 1 km spatial resolution (aggregated from the original 30 m resolution data). Elevation data were obtained from the same platform and consist of the Digital Elevation Model (DEM) product of China, also provided by the Resource and Environment Science and Data Center, Chinese Academy of Sciences (RESDC, CAS), with a spatial resolution of 30 m × 30 m; the DEM was resampled to 1 km × 1 km raster layer to match the scale of the LULC data.

#### 2.3.3 Model Construction and Calibration

##### 2.3.3.1 MaxEnt Model Construction and Optimization

We employed MaxEnt version 3.4.4 (Phillips et al. 2006, 2017) to construct a potential suitable habitat distribution model for the Oriental Stork. MaxEnt estimates species presence probability by maximizing entropy using occurrence records and environmental variables as input (Merow et al. 2013), and performs exceptionally well in predicting habitat suitability using only presence data (Elith et al. 2006; Ejigu and Tassie 2020).

To improve prediction accuracy, we systematically optimized the two core parameters—Feature Classes (FC) and Regularization Multiplier (RM)—using the “kuenm” package (Cobos et al. 2019) in R (version 4.4.3). FC includes five basic feature types: linear (L), quadratic (Q), product (P), threshold (T), and hinge (H). By exploring 31 feature combinations and 40 RM values from 0.1 to 4.0 (in steps of 0.1), a total of 1240 candidate models were generated. Using the “OR_AICc” criterion (Cobos et al. 2019), we evaluated predictive performance based on omission rate (≤ 5%). Among models meeting this criterion, complexity was further assessed using AICc, and models with ΔAICc ≤ 2 were selected as optimal.

For model operation, occurrence records and environmental layers were imported into MaxEnt. To characterize the available environmental space, 10,000 background points were randomly generated within the study extent. Occurrence data were randomly partitioned into training (75%) and testing (25%) sets. Bootstrap resampling was employed with a random seed, repeated 10 times, and the average of the 10 runs was used for subsequent analyses to evaluate model stability and reduce uncertainty. Advanced settings were as follows: maximum iterations = 500; “Write plot data” enabled; logistic output format selected to reflect species presence probability (0–1) (Merow et al. 2013); other parameters left at default.

Variable importance was assessed using percentage contribution and the jackknife test (Phillips and Dudik 2008), supplemented by response curve analysis. Model predictive power was evaluated using the Area Under the Curve (AUC) of the Receiver Operating Characteristic (ROC) curve (Phillips and Dudik 2008), with values closer to 1 indicating better predictive ability (Luo et al. 2021). Model performance criteria were: 0.5–0.6 = fail, 0.6–0.7 = poor, 0.7–0.8 = fair, 0.8–0.9 = good, and 0.9–1.0 = excellent, with AUC above 0.9 recommended for optimal performance (Sun et al. 2020).

##### 2.3.3.2 Construction of the HSI‐InVEST Integrated Model

Habitat quality refers to the ability of an environment to support the long‐term persistence of effective populations and stable communities, influenced by factors including geographical location, topography, climate, and human activities (Mohan and Kandya 2015). Assessing habitat quality can reflect regional biodiversity status (Hillard et al. 2017) and measure ecosystem integrity or degradation. To comprehensively evaluate habitat quality for the Oriental Stork, we adopted a multi‐model collaborative framework integrating the Habitat Suitability Index (HSI) model with the Habitat Quality (HQ) module of the InVEST model.

The HSI model constructs a habitat suitability evaluation system by integrating key environmental factors (Zhang et al. 2020). Here, elevation and land use type were selected as core indicators, with HSI evaluation rules established based on the species' ecological preferences. The suitability scores assigned to elevation ranges and land‐use types were derived primarily from expert judgment based on the known ecological requirements of the Oriental Stork, supplemented by published literature on the species' habitat preferences (Zheng et al. 2016; Zhou et al. 2025; Chen et al. 2026). The elevation thresholds (Table 2) were defined according to the species' documented distribution: the Oriental Stork is a typical lowland plain wetland species, with most occurrence records concentrated below 50 m elevation and virtually no records above 200 m. The land‐use suitability scores (Table 3) reflect the species' documented preferences for natural wetlands (marshland, lakes, mudflats, and floodplains receiving the highest scores) and its frequent use of paddy fields as foraging habitat (Zheng et al. 2016), while avoiding forested areas and human‐dominated landscapes. Elevation suitability was assessed using DEM data with a conditional function and tiered assignment (detailed in Table 2). The final HSI output is a comprehensive evaluation value (0–1) combining elevation and land use suitability, with higher values indicating better habitat suitability. Specifically, the HSI score for each grid cell was calculated as: HSI = 0.7 × *S*_LULC_ + 0.3 × S_elevation_, where S_elevation_ is the elevation suitability score (Table 2) and *S*_LULC_ is the land‐use suitability score (Table 3). The 7:3 weighting was chosen because land‐use type directly determines the availability of foraging, nesting, and roosting habitats, while elevation serves primarily as a macro‐scale limiting factor. It is important to clarify that the HSI and InVEST models are not integrated at the algorithmic or code level; rather, they are combined through a spatial overlay of their respective outputs. The HSI model was specifically used to compensate for the InVEST model's inherent inability to incorporate topographic factors—particularly elevation. This spatial overlay approach allows the two models to serve complementary roles in identifying priority conservation areas.

**TABLE 2:** Elevation suitability assignment rules for *Ciconia boyciana* .

Elevation range (m)	Suitability score
< −50	0.1
−50 to −20	0.2
−20 to −5	0.4
−5 to 0	0.8
0 to 50	1
50 to 200	0.65
200 to 500	0.35
500 to 1000	0.2
1000 to 2000	0.1
≥ 2000	0.05

**TABLE 3:** LULC suitability assignment rules for *Ciconia boyciana* .

LULC type	Description	Suitability score
1	Paddy field	0.7
2	Forest land	0.3
3	Grassland	0.6
4	River and canal	0.8
5	Lake	0.9
6	Reservoir and pond	0.8
7	Permanent glacier	0.05
8	Mudflat	0.9
9	Floodplain	0.8
10	Construction land	0.05
11	Sandy land	0.1
12	Gobi	0.05
13	Saline‐alkali land	0.2
14	Marshland	1
15	Bare land	0.1
16	Bare rock	0.05
17	Other land	0.1

The Habitat Quality (HQ) module of the InVEST model assesses regional habitat quality based on Land Use and Land Cover (LULC) data by quantifying the degradation effects of threat sources on habitat patches and the suitability of the habitats themselves (Jeong et al. 2024). It evaluates the sensitivity of various habitat types to anthropogenic threats (e.g., industrial activities, transportation networks, urban development), and its output serves as an effective indicator for measuring biodiversity. In this study, seven core anthropogenic threat factors were selected: paddy fields, dry farmland, urban land, rural settlements, other construction land, railways, and highways. Each threat factor was represented as a binary raster layer, with areas where the threat is present assigned a value of 1 and other areas assigned 0. All data were uniformly clipped to a 1 km × 1 km raster format. The impact intensity of a threat factor is determined by three elements: (1) the distance from the threat source to the target grid cell; (2) the relative weight of the threat; and (3) the relative sensitivity of the habitat type to that threat (Terrado et al. 2016). The weight of each threat factor (range: [0, 1]) reflects its disturbance intensity on surrounding habitats, with higher values indicating stronger disturbance. The sensitivity value (range: [0, 1]) characterizes the response degree of different land use types to ecological threats, with values closer to 1 indicating higher sensitivity (Wu et al. 2020). Following the InVEST user's guide and relevant studies, we systematically constructed a weight‐spatial decay matrix for threat factors (Table S1) and a habitat sensitivity parameter system (Table S2), assigning detailed values for weights, maximum impact distances, and spatial decay types (linear or exponential).

$$Q_{\mathrm{xj}}=H_{j}\;\left[\;1-\left(\;\frac{D_{\mathrm{xj}}^{z}}{D_{\mathrm{xj}}^{z}+k^{2}}\;\right)\right]$$

where *x*_*j*_ represents the habitat quality of grid cell *z* within habitat type *j* (range 0–1), with values closer to 1 indicating higher habitat quality; *H*_*j*_ represents habitat suitability; *K* is the half‐saturation parameter; and *D*_*xj*_ represents the degree of habitat degradation. Following model defaults and relevant research (Bai et al. 2022), the model default value of 0.5. We then ran the InVEST habitat quality module with this parameter setting and obtained the degradation summary raster, which was used as the basis for subsequent habitat quality calculations. The optimized half‐saturation parameter *K* = 0.15 was deemed appropriate for this study after examining the distribution of the resulting habitat quality scores.

The habitat quality index from the integrated model ranges from 0 to 1, with higher values indicating greater suitability as priority conservation habitat for the Oriental Stork. Based on existing research and study objectives, habitat quality was classified into four tiers: high (≥ 0.8), medium (0.65–0.8), relatively low (0.4–0.65), and low (< 0.4). Areas classified as high (≥ 0.8) were designated as core habitat zones.

### 2.4 Suitable Distribution Area

Based on the continuous raster data in logistic format output by MaxEnt, we conducted post‐processing and spatial analysis using ArcGIS 10.8. To characterize the spatial distribution pattern of habitat suitability, a manual classification method was used to divide the probability values (0–1) into four levels: unsuitable (< 0.2), low suitability (0.2–0.4), moderate suitability (0.4–0.6), and high suitability (> 0.6). Final thresholds were determined by averaging results from 10 bootstrap replicates to ensure robustness.

To assess dynamic changes in suitable habitats under future climate scenarios, we uniformly converted suitability distribution maps for current and future periods (SSP1‐2.6, SSP2‐4.5, SSP5‐8.5) into binary format, classifying areas as “suitable” (≥ 0.2) or “unsuitable” (< 0.2). Using the “Distribution Changes Between Binary SDMs” module in SDM Toolbox v2.6, habitat dynamics were quantitatively analyzed and classified into three types: (1) increase (unsuitable to suitable); (2) stable (suitable in both periods); and (3) decrease (suitable to unsuitable). This quantified changes in suitable habitat area and spatial pattern shifts under different climate scenarios.

To further reveal spatial migration trends, we used the geosphere package (version 1.5‐20) in R to calculate the geometric center (centroid) of suitable distribution areas under different periods and scenarios. Migration distance and direction were used to characterize spatial pattern changes. By integrating distribution probability patterns with centroid migration trajectories, we systematically evaluated the spatiotemporal dynamics of suitable distribution areas for the Oriental Stork under different climate scenarios.

### 2.5 Multivariate Environmental Similarity Surface and Most Dissimilar Variables Analysis

Using current climate layers as a reference (with current suitable area delineated in ArcGIS), we applied the Multivariate Environmental Similarity Surface (MESS) method to assess potential impacts of future climate scenarios on habitat suitability. MESS quantifies similarity between future and current climates for each area, where *S* = 100 indicates complete identity with the reference, and *S* < 0 indicates at least one bioclimatic variable falls outside the reference range, representing novel climate conditions. The most dissimilar variable (MOD) was also identified to pinpoint key drivers of distribution changes.

For the 10 replicate runs, the Cell Statistics tool in ArcGIS was used to calculate the mean MESS raster for each scenario. Mean MESS values were reclassified into five risk levels: severe extrapolation (< −50), moderate extrapolation (−50 to −11), minor extrapolation (−10 to −1), marginal within‐range (0 to 50), and typical within‐range (> 50) to quantify spatial distribution and area proportions of extrapolation degrees.

For MOD analysis, all threshold features were aggregated by their derived source and merged into corresponding original variables to facilitate ecological interpretation. The Cell Statistics tool was used to calculate the mode of MOD results from the 10 replicates, representing the most stable dominant extrapolation variable for each grid cell. Occurrence frequency and spatial distribution of each variable within extrapolation areas were statistically analyzed using the Density and Novel tools in MaxEnt (Elith et al. 2010).

### 2.6 Identification of Priority Conservation Areas and Conservation Gap Assessment

To accurately identify core conservation areas for the Oriental Stork, we conducted a multi‐model spatial overlay analysis in ArcGIS 10.8, integrating the habitat suitability map from MaxEnt with habitat quality results from the HSI‐InVEST model. Through raster overlay calculation and spatial registration, spatial units meeting dual screening criteria—“dual‐high areas”—were extracted as priority conservation areas, defined as areas with medium or high suitability (≥ 0.4) from MaxEnt and high‐quality habitat (habitat quality index ≥ 0.8) from HSI‐InVEST. All raster data were preprocessed in ArcGIS 10.8: uniformly projected to WGS 1984 (EPSG: 4326), resampled to a unified cell size of 2.5 arc‐minutes using the Resample tool, and clipped to China's administrative boundary. Raster alignment was verified by ensuring identical spatial extent and snap‐to‐raster consistency across all layers prior to overlay calculations.

To assess the current conservation status of these priority areas and identify conservation gaps under the “30 × 30” target, we overlaid the priority conservation areas with the existing protected area network in China. Conservation gap analysis is a widely used approach for evaluating protected area effectiveness by identifying the extent to which high‐priority habitats are covered by existing protected areas relative to conservation targets (Rodrigues, Akcakaya, et al. 2004; Wang et al. 2024). Areas identified as priority conservation areas that do not overlap with existing protected areas are defined as unprotected priority areas (conservation gaps). For a given species, if the proportion of its predicted priority distribution area covered by protected areas meets the conservation target, the protection is considered effective; conversely, if the coverage falls below the target, a conservation gap exists (Rodrigues, Andelman, et al. 2004; Wang et al. 2024). The protected area data used in this study, covering 1028 nature reserves and 8 national parks for the year 2020, were obtained from the Resource and Environment Science and Data Platform (RESDC, https://www.resdc.cn/).

This identification method is based on a multi‐model collaborative verification framework that combines the strengths of MaxEnt and HSI‐InVEST to achieve a comprehensive determination of priority areas. Validated through this dual approach, these areas provide optimal habitat conditions essential for the survival and reproduction of the Oriental Stork while maintaining key ecosystem functions and services, offering a scientific basis for formulating targeted zoning conservation strategies and spatial management measures. For the “30 × 30” target assessment, the additional area required to meet the representation target was calculated as: (Total priority habitat area × 30%) − Protected priority habitat area. This calculation distinguishes total priority habitat, protected priority habitat, unprotected priority habitat, and the additional area needed to achieve the 30% representation goal. The gap analysis results serve as a quantitative basis for recommending priority areas for protected area expansion and for evaluating progress toward the “30 × 30” target.

## 3 Results

### 3.1 MaxEnt Model Optimization Results

Based on systematic optimization using the “kuenm” package, the model performed optimally with feature combination set to Linear and Threshold (LT) and regularization multiplier set to 1.6, significantly outperforming other candidate parameter combinations (Figure 4). Under this optimal configuration, the mean AUC from 10 replicate runs reached 0.943, the 5% training omission rate was 4.79%, and ΔAICc = 0. These results indicate that the model possesses excellent predictive accuracy, robustness, and generalization ability, making it suitable for subsequent predictions of species potential distribution.

**FIGURE 4:**
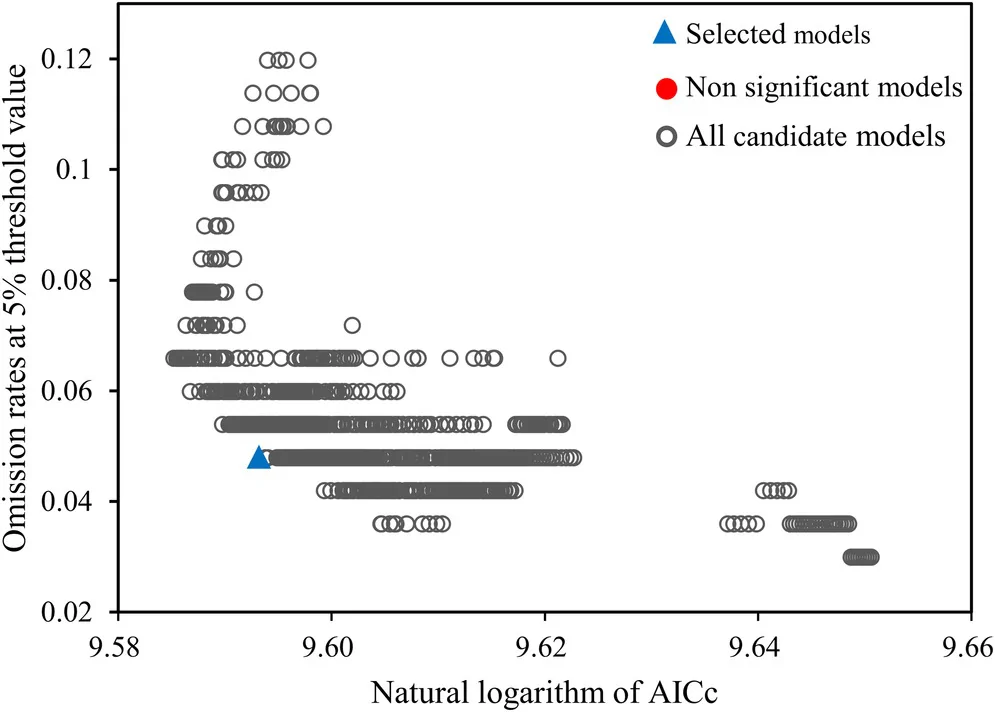
Two‐step model selection process for MaxEnt calibration: Candidate models were first filtered by omission rate (≤ 5%), and subsequently by ΔAICc (≤ 2) to identify the optimal parameter set.

### 3.2 Current Suitable Distribution Area

MaxEnt model predictions were visualized in ArcGIS to present the suitability distribution of the Oriental Stork under current climatic conditions (Figure 5). The species is mainly distributed in eastern China, including Heilongjiang, Hebei, Shandong, Jiangsu, Hunan, and Guangdong. The total suitable habitat area is approximately 106.57 × 10^4^ km^2^, accounting for 11.1% of China's terrestrial area. The highly suitable area is approximately 9.49 × 10^4^ km^2^ (0.99% of China's total terrestrial area), mainly distributed in the southern Northeast Plain (south‐central Liaoning), the North China Plain (Beijing, Tianjin, Hebei, Shandong), the Middle‐Lower Yangtze River Plain (Shanghai, Jiangsu, Zhejiang, Anhui, Hubei, Hunan, Jiangxi), the Pearl River Delta (Guangdong), and the western plain of Taiwan. Overall, suitable distribution areas are concentrated in the Northeast Plain, North China Plain, Huang‐Huai‐Hai Plain, Middle‐Lower Yangtze River Plain, and coastal areas of South China. The predictions are highly consistent with the known geographical distribution of the Oriental Stork, indicating high model reliability.

**FIGURE 5:**
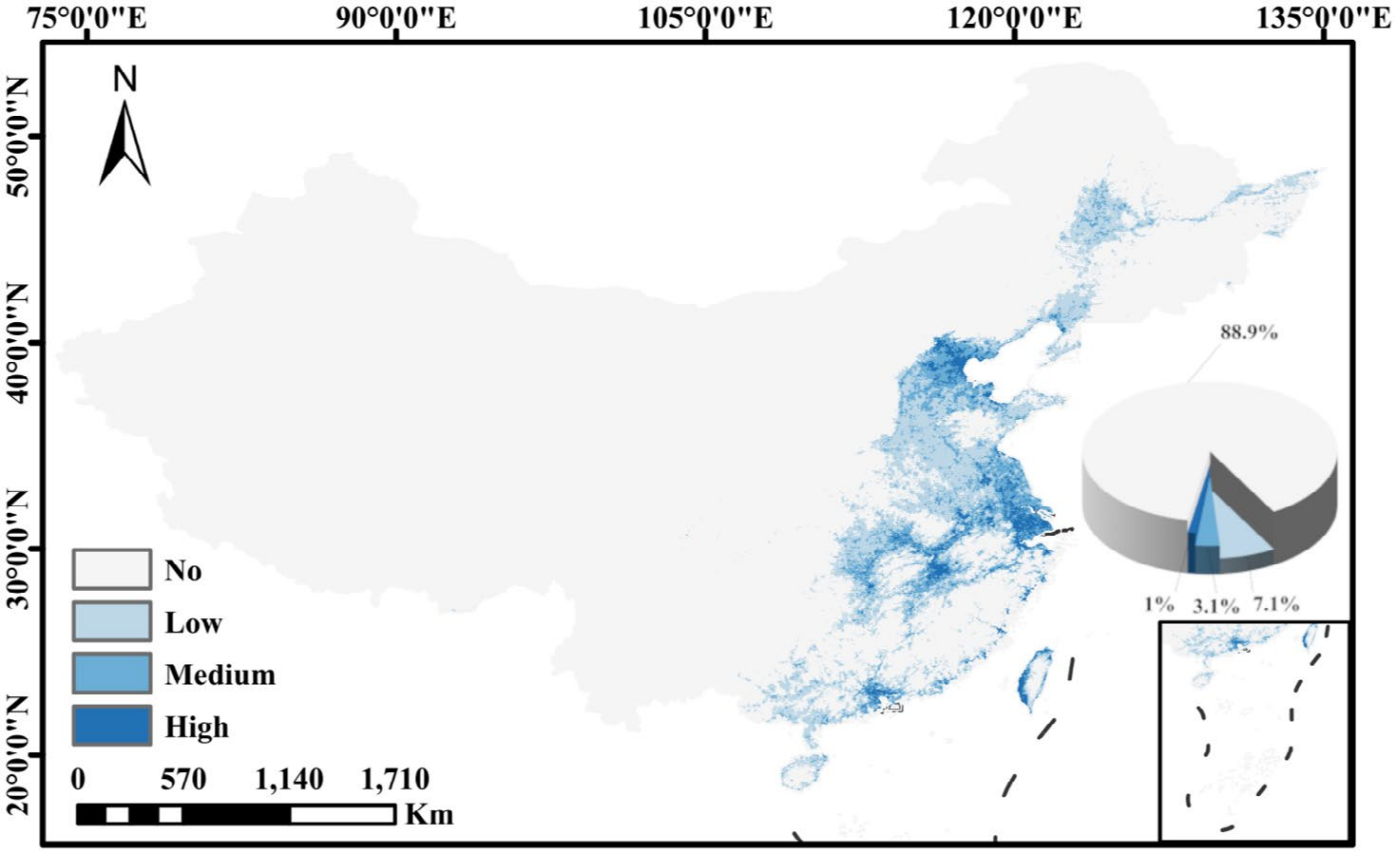
Current prediction of suitable distribution areas for *Ciconia boyciana* .

### 3.3 Future Suitable Distribution Area

Future suitable area distribution maps for the Oriental Stork were generated using ArcGIS 10.8 (Figure 6). Under different climate scenarios, the potential suitable habitat area is projected to show a general expansion trend, though the magnitude varies across scenarios and time periods (Table 4). The most pronounced expansion occurs under SSP5‐8.5 during 2061–2080: total suitable area increases from 106.57 × 10^4^ km^2^ to 114.90 × 10^4^ km^2^ (+8.33 × 10^4^ km^2^, 7.82%). The smallest expansion is under SSP2‐4.5 during 2041–2060: total suitable area reaches 106.80 × 10^4^ km^2^ (+0.23 × 10^4^ km^2^, 0.22%).

**FIGURE 6:**
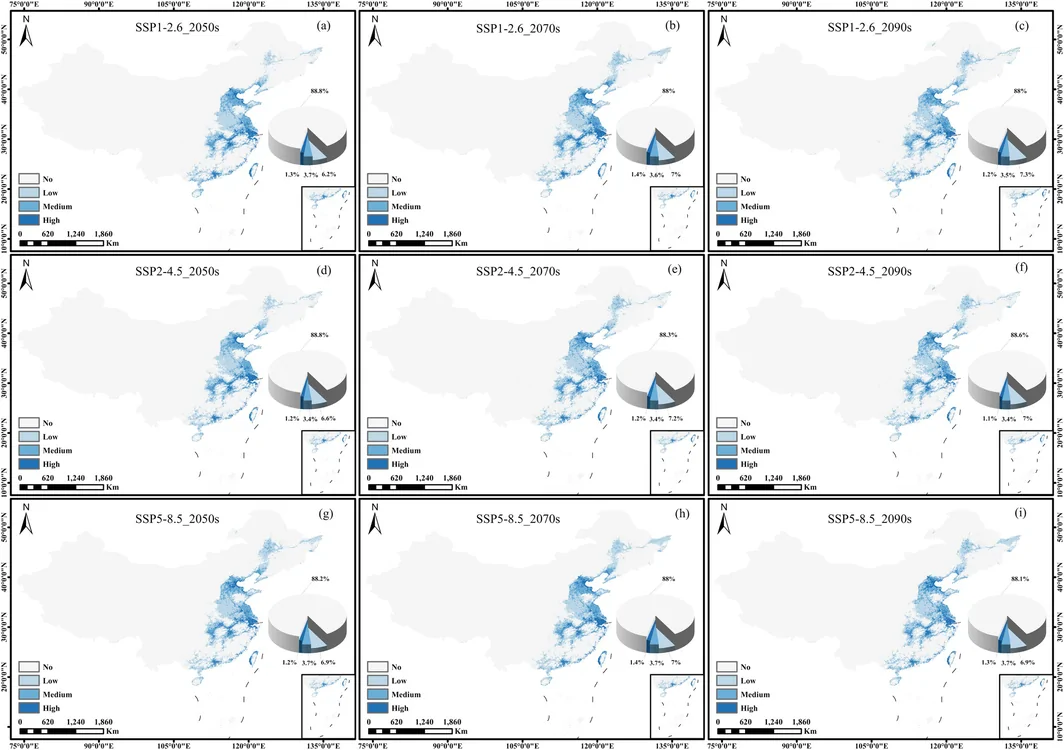
Prediction of suitable distribution areas for *Ciconia boyciana* in the future. (a) SSP1‐2.6 in the 2050s; (b) SSP1‐2.6 in the 2070s; (c) SSP1‐2.6 in the 2090s; (d) SSP2‐4.5 in the 2050s; (e) SSP2‐4.5 in the 2070s; (f) SSP2‐4.5 in the 2090s; (g) SSP5‐8.5 in the 2050s; (h) SSP5‐8.5 in the 2070s; (i) SSP5‐8.5 in the 2090s.

**TABLE 4:** Suitable distribution area under different climatic scenarios.

Climate scenario	Predicted area (10^4^ km^2^)				Change rate (%)
	High	Medium	Low	Total	
Current	9.49	29.37	67.71	106.57	
SSP126_2050S	12.48	35.39	59.55	107.42	0.80
SSP126_2070S	13.26	34.30	67.05	114.61	7.55
SSP126_2090S	11.19	33.56	70.21	114.96	7.88
SSP245_2050S	11.22	32.22	63.36	106.80	0.22
SSP245_2070S	11.19	32.35	68.86	112.40	5.48
SSP245_2090S	10.16	32.62	66.60	109.38	2.64
SSP585_2050S	11.40	35.12	66.05	112.57	5.64
SSP585_2070S	12.93	35.44	66.53	114.90	7.82
SSP585_2090S	12.28	35.50	66.48	114.26	7.22

Changes in high, medium, and low suitability areas vary across scenarios. Highly suitable areas expand most under SSP1‐2.6 during 2061–2080 (9.49 to 13.26 × 10^4^ km^2^). Medium suitability peaks under SSP5‐8.5 during 2081–2100 (35.50 × 10^4^ km^2^), and low suitability peaks under SSP1‐2.6 during 2081–2100 (70.21 × 10^4^ km^2^). Overall, the potential suitable habitats may exhibit varying degrees of response to climate change across scenarios, reflecting the species' sensitivity to future climatic changes.

### 3.4 Dynamic Changes in Suitable Areas Across Different Periods

Compared to the current situation, suitable distribution areas vary across climate scenarios and time periods (Table 5 and Figure 7). Under SSP1‐2.6 during 2080–2100, the decrease in suitable area is smallest (1.43 × 10^4^ km^2^, 1.34%), while under SSP1‐2.6 during 2060–2080, the decrease is largest (5.01 × 10^4^ km^2^, 4.70%). The most significant increase occurs under SSP1‐2.6 during 2060–2080 (10.39 × 10^4^ km^2^, 9.75%), and the smallest increase under SSP2‐4.5 during 2040–2060 (5.62 × 10^4^ km^2^, 5.27%).

**TABLE 5:** Current and future changes in suitable distribution areas for *Ciconia boyciana* .

Climate scenario	Suitable distribution area (10^4^ km^2^)	Increase (10^4^ km^2^)	Stable (10^4^ km^2^)	Decrease (10^4^ km^2^)	Percentage gain (%)	Percentage loss (%)
Current	106.57	—	—	—	—	—
SSP126_2050S	107.42	6.68	103.99	5.01	6.27	4.70
SSP126_2070S	114.61	10.39	106.54	2.46	9.75	2.31
SSP126_2090S	114.96	9.89	107.57	1.43	9.28	1.34
SSP245_2050S	106.80	5.62	104.30	4.70	5.27	4.41
SSP245_2070S	112.40	8.72	106.39	2.61	8.18	2.45
SSP245_2090S	109.38	6.01	105.93	3.07	5.64	2.88
SSP585_2050S	112.57	8.87	105.77	3.23	8.32	3.03
SSP585_2070S	114.90	10.37	106.71	2.29	9.73	2.15
SSP585_2090S	114.26	9.68	106.68	2.33	9.08	2.19

**FIGURE 7:**
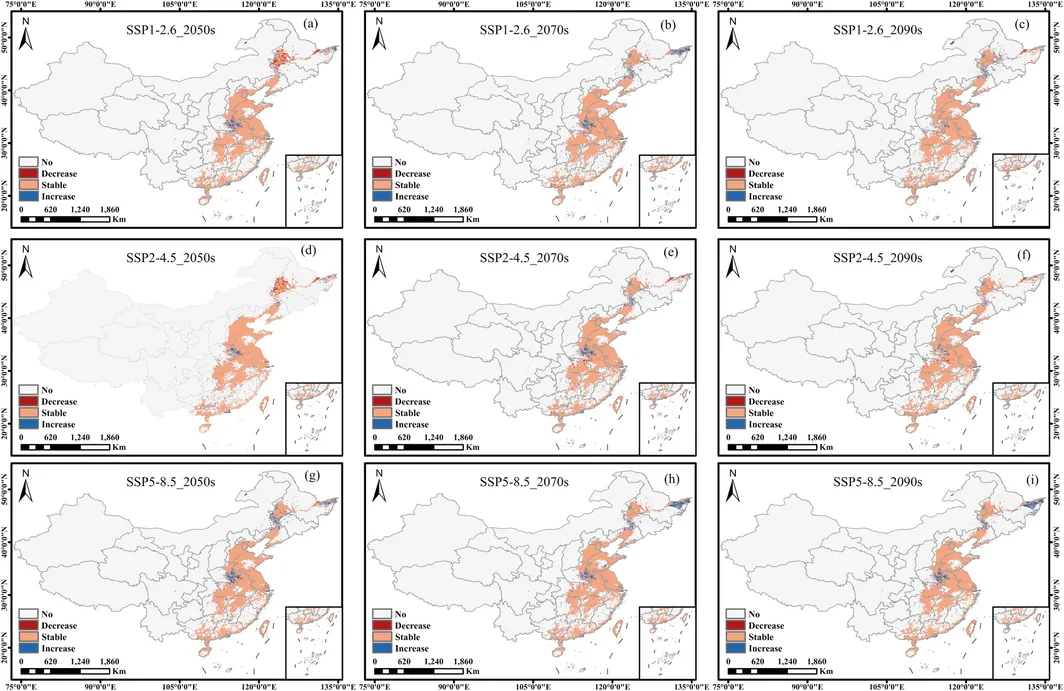
Dynamic changes of *Ciconia boyciana* under future climate conditions. (a) SSP1‐2.6 in the 2050s; (b) SSP1‐2.6 in the 2070s; (c) SSP1‐2.6 in the 2090s; (d) SSP2‐4.5 in the 2050s; (e) SSP2‐4.5 in the 2070s; (f) SSP2‐4.5 in the 2090s; (g) SSP5‐8.5 in the 2050s; (h) SSP5‐8.5 in the 2070s; (i) SSP5‐8.5 in the 2090s.

As shown in Figure 7, areas with no change in suitability are mainly distributed across Beijing, Tianjin, Hebei, Shandong, Jiangsu, Shanghai, Zhejiang, Anhui, and Hubei. Areas with decreased suitability are concentrated in south‐central Liaoning, central Shandong, and northern Jiangsu, while areas with increased suitability are primarily located in Heilongjiang, Jilin, eastern Inner Mongolia, and Taiwan. Overall, habitat suitability shows a significant response to climate change, with consistent trends across similar scenarios. Although all future scenarios indicate an overall expansion trend, the magnitude varies: SSP5‐8.5 shows the most rapid early expansion, SSP1‐2.6 maintains relatively high increases in the middle and late periods, while SSP2‐4.5 exhibits smaller expansion across all periods with a later decline.

From a spatial perspective (Figure 8), the habitat centroid generally shows a projected northeastward shift. Under current climate, the centroid is located near Guzhen County, Anhui (117°44′34″ E, 33°47′42″ N). Under different scenarios and periods, projected centroid displacements range from 16.82 to 77.22 km, with the largest under SSP5‐8.5 during 2041–2060 (77.22 km northeast). These results indicate that future changes in precipitation and temperature are projected to drive the northeastward shift of the centroid of potentially suitable habitat.

**FIGURE 8:**
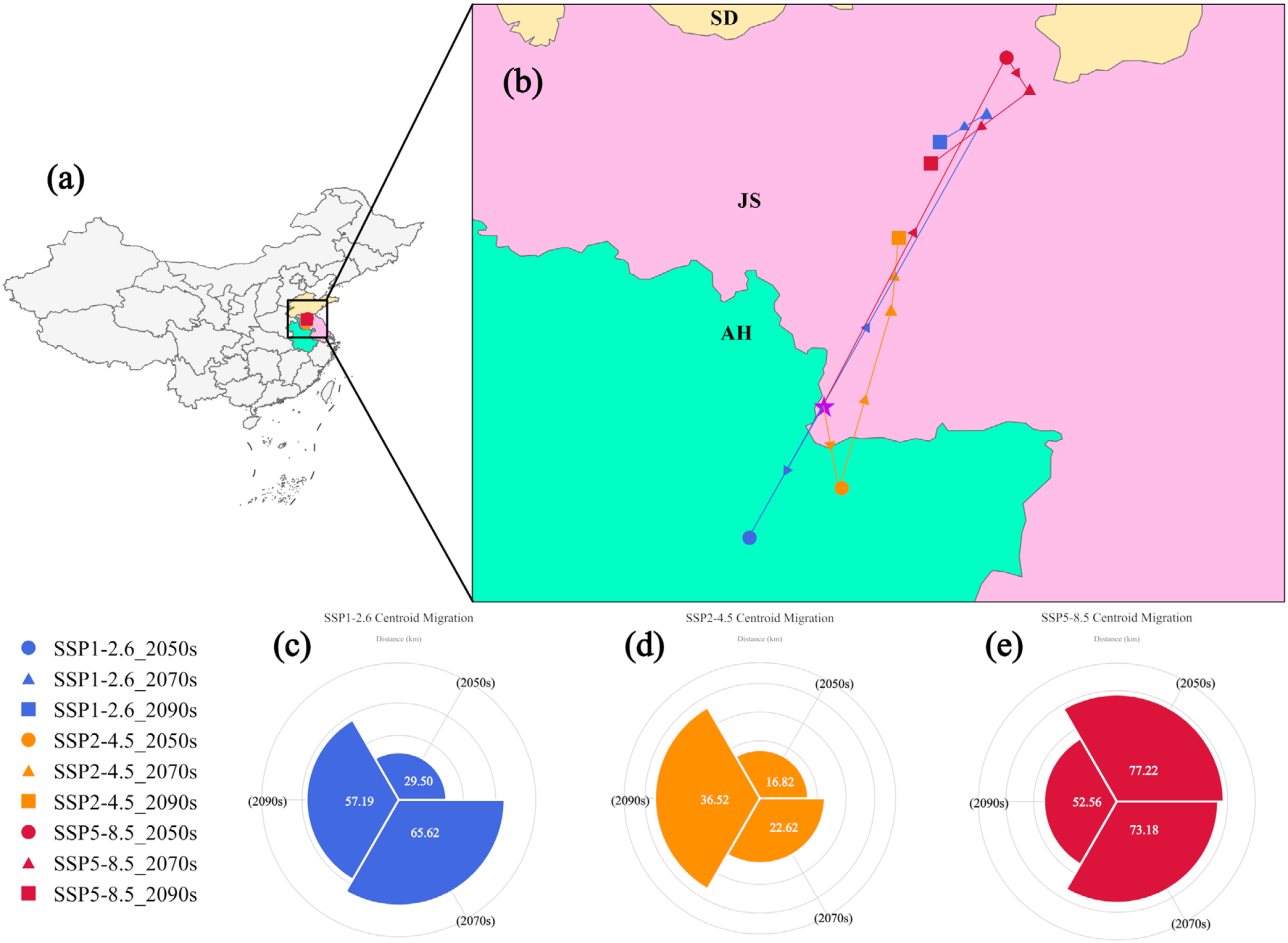
Geographical changes of the central particle in *Ciconia boyciana* under different climatic scenarios and periods [(b) is an enlargement of the part of (a); (c) centroid migration distance of *Ciconia boyciana* under SSP1‐2.6; (d) centroid migration distance of *Ciconia boyciana* under SSP2‐4.5; (e) centroid migration distance of *Ciconia boyciana* under SSP5‐8.5].

### 3.5 Multivariate Environmental Similarity Surface and Most Dissimilar Variable Analysis

Based on the mean MESS from 10 replicate runs (Figure 9), the average multivariate environmental similarity values under future climate scenarios range from 9.50 to 12.70, indicating that future conditions maintain some similarity with current climate (MESS > 0). During 2041–2060, SSP1‐2.6 shows the highest mean MESS (12.70, lowest climate anomaly), while SSP5‐8.5 shows the lowest (11.10). During 2061–2080, SSP1‐2.6 again has the highest (11.16) and SSP5‐8.5 the lowest (9.50). Over time and with emission intensification, MESS values show a decreasing trend, indicating progressively diminishing similarity. Notably, under SSP5‐8.5 in the 2090s, MESS (9.50) approaches the low similarity threshold (Figure 9).

**FIGURE 9:**
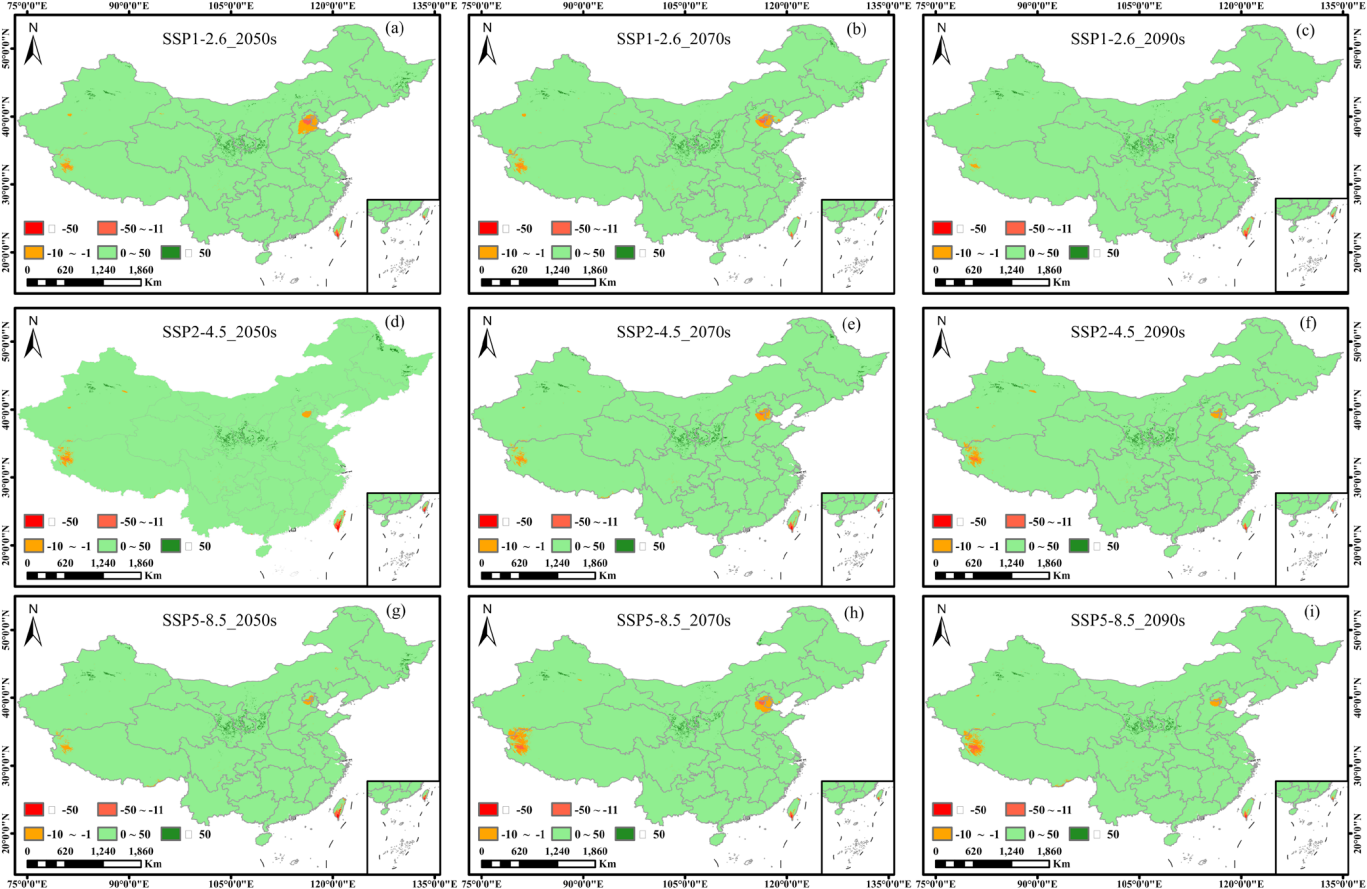
Multivariate environmental similarities of *Ciconia boyciana* . (a) SSP1‐2.6 in the 2050s; (b) SSP1‐2.6 in the 2070s; (c) SSP1‐2.6 in the 2090s; (d) SSP2‐4.5 in the 2050s; (e) SSP2‐4.5 in the 2070s; (f) SSP2‐4.5 in the 2090s; (g) SSP5‐8.5 in the 2050s; (h) SSP5‐8.5 in the 2070s; (i) SSP5‐8.5 in the 2090s.

Following the five‐level classification criteria (Elith et al. 2010), “marginal within‐range areas” (0–50) were the dominant type across all scenarios, accounting for 97.76%–98.85% of the study area. The SSP1‐2.6_2090s scenario had the highest proportion (98.85%), while SSP5‐8.5_2070s had the lowest (97.76%). Overall, MESS values remained high (> 97%) across all scenarios, indicating that most areas retain similarity with the model training range, resulting in low extrapolation uncertainty and good prediction reliability. Areas classified as “severe extrapolation” (< −50) accounted for less than 0.04%, mainly concentrated in southwestern Xinjiang (Tarim Basin), western Tibet, and eastern Taiwan Island (Figure 9).

The Most Dissimilar Variable (MOD) analysis revealed key drivers of environmental extrapolation (Figure 10). Based on modal MOD results from 10 replicates, precipitation seasonality (Bio15) and precipitation of the wettest month (Bio13) were identified as the primary variables. Bio15 exhibited the most significant dominant effect across all periods, with contribution proportions ranging from 63.93% to 92.58%, mainly distributed in the eastern monsoon region (North China Plain, Northeast Plain, Middle‐Lower Yangtze River Plain) and margins of the Qinghai‐Tibet Plateau. Bio13 served as the secondary dominant variable, mainly concentrated in southern Taiwan and southwestern Xinjiang. Although mean diurnal temperature range (Bio2) did not become a dominant variable at the full scale, it emerged as a local limiting factor in specific scenarios (e.g., SSP1‐2.6_2090s, SSP5‐8.5_2090s) (Figure 10). By factor category, precipitation‐related variables contributed the most (92.55% of total extrapolation area: Bio15 79.24%, Bio13 12.35%, Bio19 0.96%), followed by topography (4.52%) and temperature (1.85%).

**FIGURE 10:**
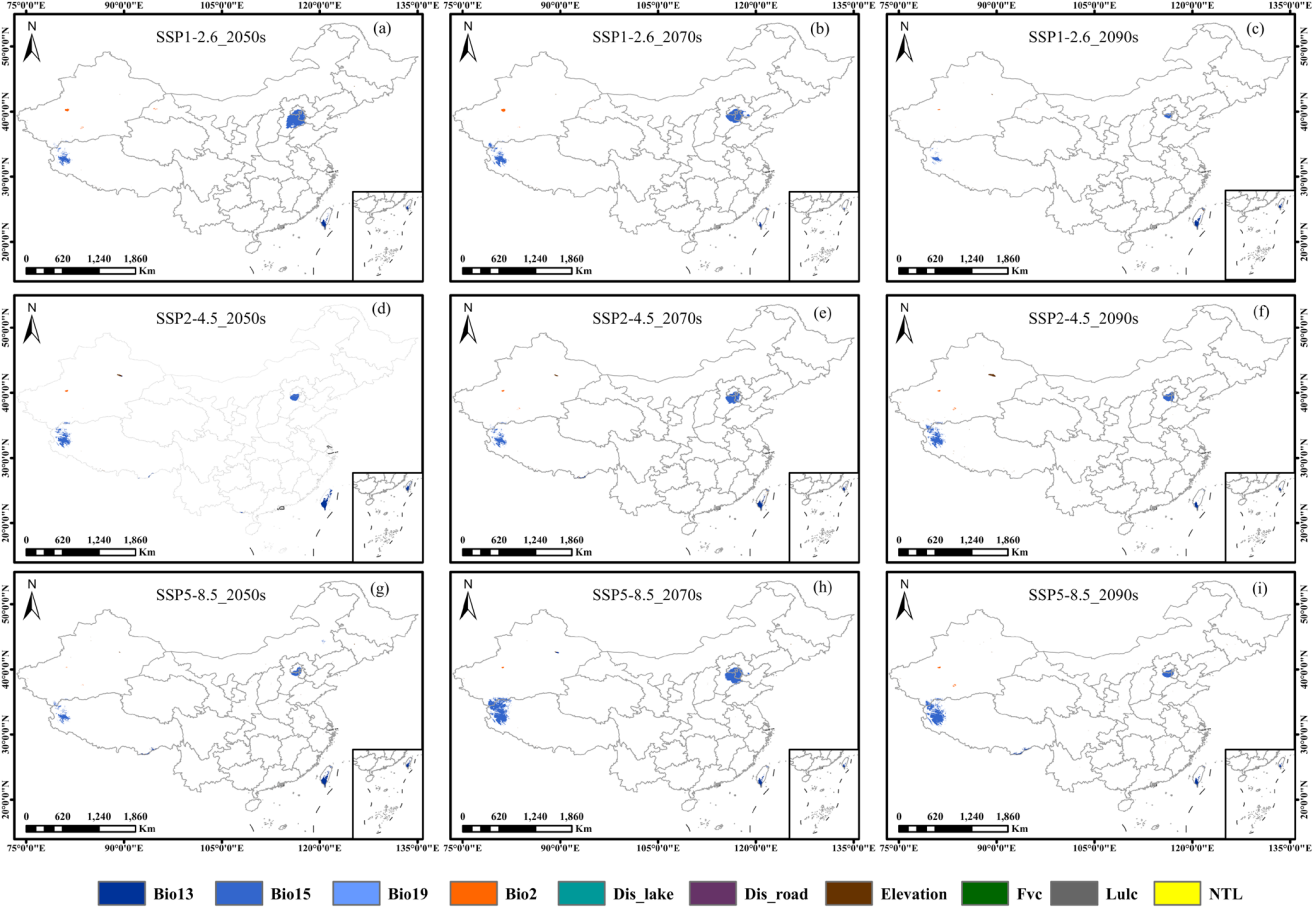
Most dissimilar variables of *Ciconia boyciana* . (a) SSP1‐2.6 in the 2050s; (b) SSP1‐2.6 in the 2070s; (c) SSP1‐2.6 in the 2090s; (d) SSP2‐4.5 in the 2050s; (e) SSP2‐4.5 in the 2070s; (f) SSP2‐4.5 in the 2090s; (g) SSP5‐8.5 in the 2050s; (h) SSP5‐8.5 in the 2070s; (i) SSP5‐8.5 in the 2090s.

### 3.6 The Main Environmental Factors Affecting the Distribution of *Ciconia boyciana*

According to MaxEnt model predictions, the six dominant variables influencing Oriental Stork distribution are, in order: altitude (80.3%), bio13 (5.1%), distance to lakes (3.8%), distance to roads (2.9%), bio2 (2.1%), and bio19 (1.6%). In the jackknife test (Figure 11), altitude exhibited the highest normalized training gain when used in isolation and the lowest gain when excluded, highlighting its unique importance.

**FIGURE 11:**
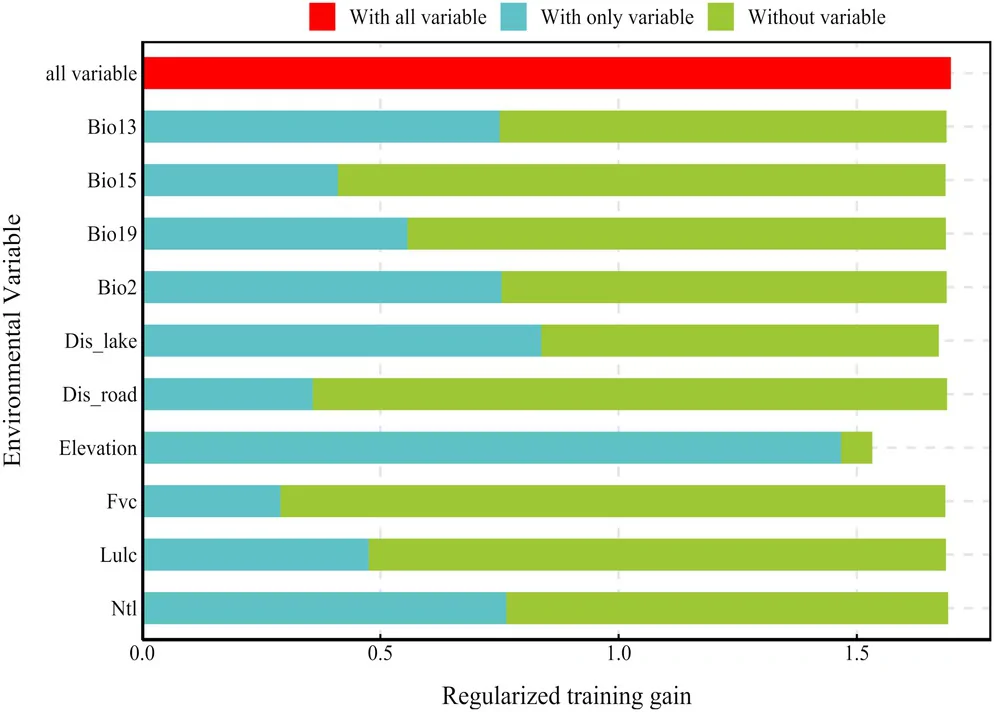
Regularized training gain (Green represents without variable, blue represents without only variable, and red represents all variables).

The response curves for these six variables (Figure 12) indicate that the range where probability of presence exceeds 0.5 is considered suitable habitat. Altitude is the most influential factor: the predicted probability remains above 0.68 below 11.7 m and drops below 0.5 at 26.9 m. The suitable range is reported as −3.5 to 11.7 m, where −3.5 m represents the lowest reliable data point available in the elevation response curve, and 11.7 m is the upper limit precisely determined via linear interpolation between adjacent response‐curve data points using the threshold method (probability ≥ 0.5). The value at 26.9 m is an actual sampled point on the response curve that falls below the 0.5 threshold, illustrating the continuous decline in suitability with increasing elevation. For distance to lake, two suitable intervals were identified: a near‐lake interval (0.003–0.041 km) and a far‐lake interval (1.47–3.55 km). The suitable range for distance to road is > 1.2576 km. For bioclimatic variables, suitable ranges are: mean diurnal temperature range (bio2) 1.52°C–8.76°C, precipitation of the wettest month (bio13) 189.3–976.4 mm, and precipitation of the coldest quarter (bio19) 83.3–790.2 mm.

**FIGURE 12:**
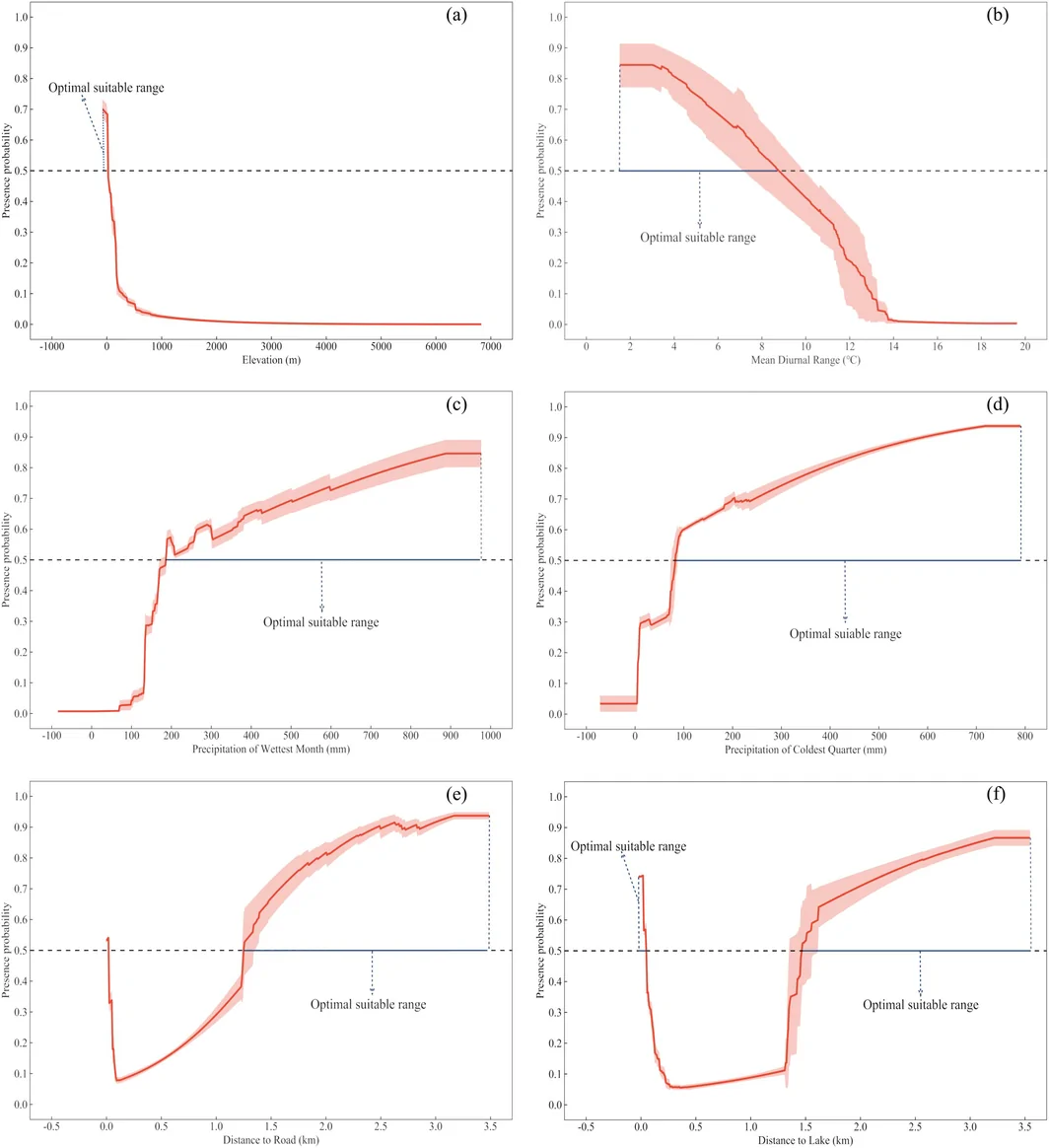
Response curves of the current climate [(a) elevation, (b) bio2, (c) bio13, (d) bio19, (e) distance to road, (f) distance to lake].

### 3.7 Priority Conservation Areas and Conservation Gaps

We used the HSI‐InVEST integrated model to calculate habitat quality for the Oriental Stork (Figure 13) and imported the results into ArcGIS. The HSI model, constructed based on land use and altitude factors with a 7:3 weight configuration, balanced the limiting effect of altitude with the influence of land use types on habitat suitability. The InVEST model effectively revealed the spatial impact pattern of human activity threats on habitat quality, enhancing the integrated model's responsiveness to anthropogenic disturbance.

**FIGURE 13:**
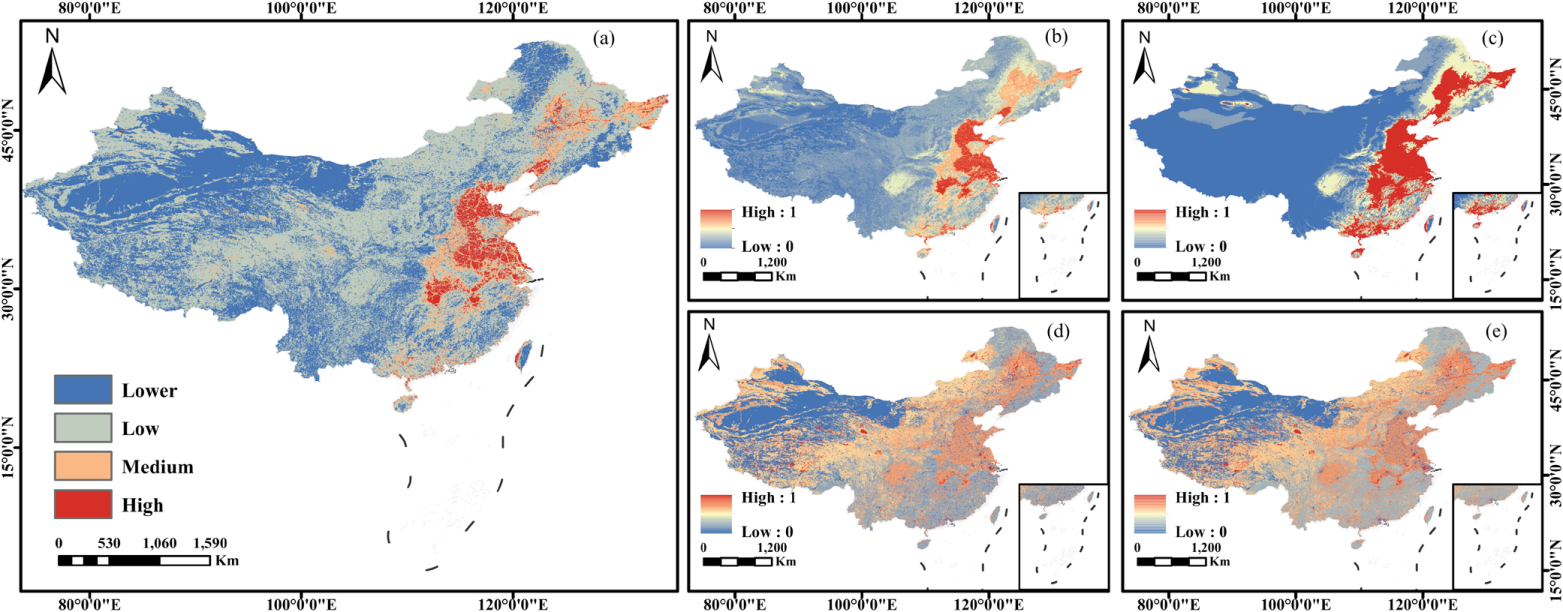
Habitat quality of *Ciconia boyciana* in China [(a) HSI‐InVEST integrated model, (b) HSI model, (c) Elevation suitability for *Ciconia boyciana* , (d) InVEST‐HQ model. (e) Land use suitability for *Ciconia boyciana* ].

Habitat quality in China exhibits a significant spatial differentiation pattern characterized by “high in the east and low in the west,” with grades decreasing progressively from the southeastern coast toward the northwestern interior. High‐quality habitats are highly concentrated in eastern coastal regions, displaying a “C”‐shaped distribution pattern, primarily including areas rich in wetland resources such as Bohai Bay, the Yangtze River Delta, and the Pearl River Delta, which provide ideal foraging and breeding grounds.

As shown in Figure 14, priority conservation areas are mainly concentrated in lake beaches and floodplain wetlands of the North China Plain, Huang‐Huai‐Hai Plain, and Jianghuai region. These include: Baiyangdian wetland in south‐central Hebei and contiguous wetlands in the Beijing‐Tianjin‐Hebei region; bottomlands around Nansi Lake and Dongping Lake in western Shandong, and wetlands along the Yellow River in northwestern Shandong; Hongze Lake, Yancheng wetlands, Luoma Lake, Lixiahe wetland, and coastal tidal flats in northern Jiangsu; bottomlands along the Huaihe River in northern Anhui, wetlands around Wabu Lake, and river network wetlands in the Huaibei Plain. Priority areas are also distributed in the southern Songnen Plain, Liaohe Plain wetlands in central Liaoning, northern Jianghan Plain in eastern Hubei, wetlands around Dongting Lake in northern Hunan, areas surrounding Poyang Lake in Jiangxi, the Pearl River Delta, and coastal wetlands in western Taiwan.

**FIGURE 14:**
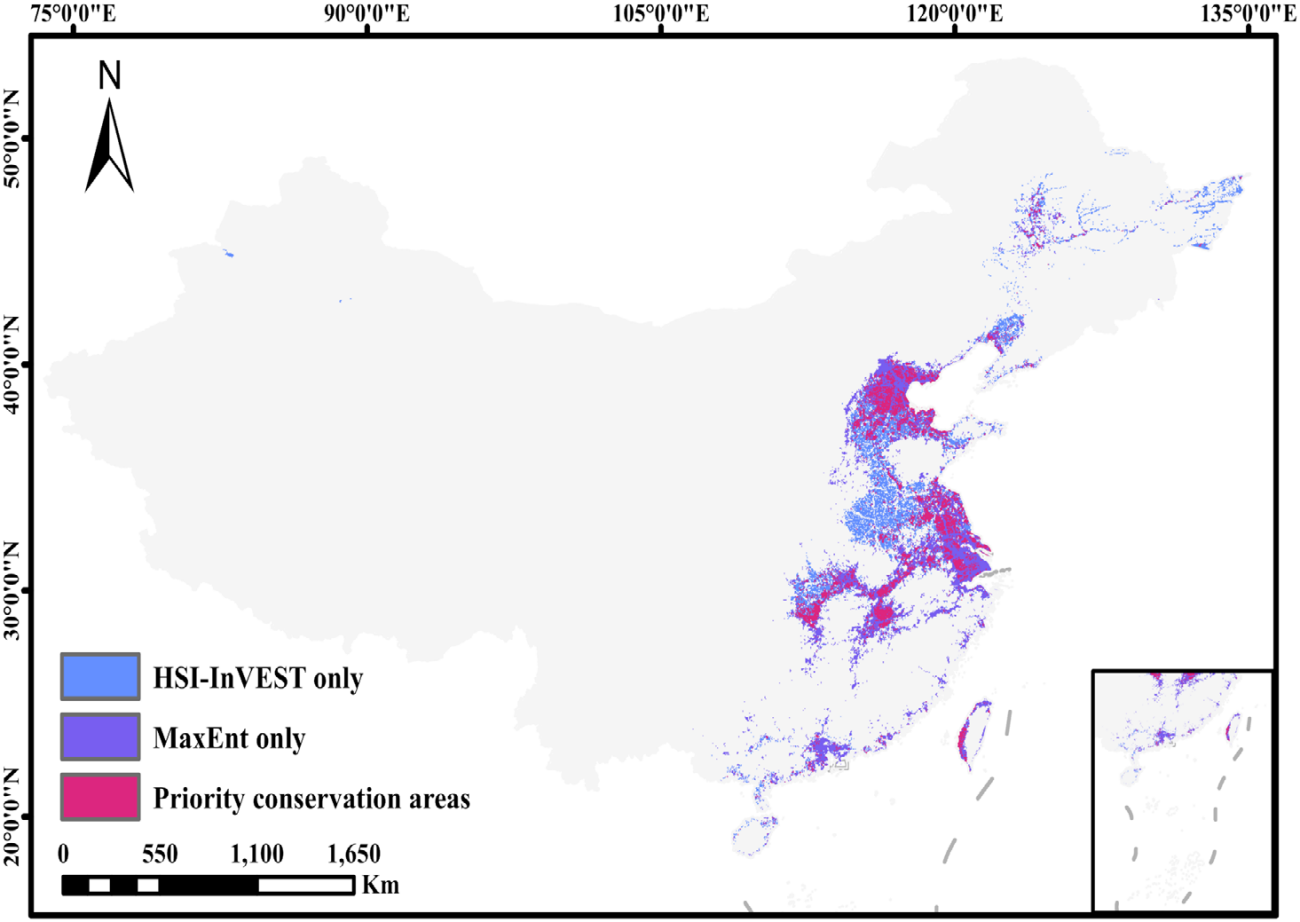
Priority conservation areas for *Ciconia boyciana* in China (Blue represents high habitat quality areas identified only by the HSI‐InVEST integrated model, purple represents highly suitable distribution areas identified only by the MaxEnt model, red represents “dual‐high areas” that meet both screening criteria).

The total area of priority conservation areas is 149,618 km^2^. To assess the current conservation status of these priority areas, we overlaid them with the existing protected area network in China. The analysis revealed that 17,170 km^2^ of the priority conservation areas currently fall within existing protected areas, while 132,448 km^2^ remain unprotected, representing significant conservation gaps. To meet the “30 × 30” representation target, the additional area requiring protection is 27,715 km^2^. These gap areas are concentrated in several key regions: (1) the Beijing‐Tianjin‐Hebei region (Baiyangdian wetland and contiguous wetlands); (2) northern Jiangsu (Hongze Lake, Yancheng wetlands, Luoma Lake, Lixiahe wetland, and coastal tidal flats); (3) northern Jianghan Plain in eastern Hubei; and (4) the areas surrounding Dongting Lake and Poyang Lake in Hunan and Jiangxi. These unprotected high‐quality habitats represent critical conservation opportunities for achieving the “30 × 30” target. The inclusion of these gap areas into the protected area network, either by designating new nature reserves or enlarging existing ones, would make a substantial contribution to the national “30 × 30” target, especially in eastern China, where protected area coverage is currently insufficient.

## 4 Discussion

### 4.1 Key Variables for the Oriental Stork

Understanding the relationship between species distribution and environmental variables is a crucial first step in conservation (Harapan et al. 2022). The MaxEnt model identified six dominant factors influencing the Oriental Stork distribution, in order: altitude (80.3%), precipitation of the wettest month (bio13, 5.1%), distance to lakes (3.8%), distance to roads (2.9%), mean diurnal temperature range (bio2, 2.1%), and precipitation of the coldest quarter (bio19, 1.6%). Topographical factors play a decisive role, while hydrological, climatic, and anthropogenic disturbance factors constitute secondary but important regulators. The jackknife test further confirmed altitude's dominant role: when used in isolation, it achieved the highest normalized training gain, and when excluded, the gain dropped to the lowest level, highlighting its irreplaceable contribution. This finding aligns with existing research on wetland bird sensitivity to topography (Zhou et al. 2025). By influencing hydrological processes, vegetation types, and human activity intensity, altitude indirectly shapes suitable habitat patterns.

Response curves reveal clear threshold characteristics for each dominant factor. For altitude, the probability of presence remains above 0.68 below 11.7 m, drops below 0.5 at 26.9 m, with the suitable range concentrated between −3.5 m and 11.7 m, indicating that the Oriental Stork is a typical lowland plain wetland species highly sensitive to elevation changes. The distance to lakes response curve exhibits a bimodal pattern, with two suitable intervals: 0.0026–0.0413 km and 1.4724–3.5456 km, which may correspond to different habitat uses such as foraging and nesting, although this functional assignment requires verification with seasonally partitioned occurrence data (Zheng et al. 2016; Zhou et al. 2025). The suitable range for distance to roads is > 1.2576 km, reflecting avoidance of human disturbance. For bioclimatic variables, suitable ranges are: mean diurnal temperature range (bio2) 1.52°C–8.76°C; precipitation of the wettest month (bio13) 189.3–976.4 mm; and precipitation of the coldest quarter (bio19) 83.3–790.2 mm. These precipitation factors reflect the species' water requirements: abundant precipitation maintains wetland hydrology and food supply, while excessive precipitation may submerge foraging grounds (He et al. 2025).

Overall, the distribution of the Oriental Stork is shaped by the combined effects of topographical dominance, hydrological regulation, and anthropogenic disturbance. Altitude delimits the macro‐geographical pattern, while distance to lakes and precipitation determine local habitat suitability, and distance to roads reflects human activity impacts. The identification of suitable ranges provides a quantitative basis for protected area delineation and habitat restoration: priority should be given to protecting lake wetlands below 11.7 m, maintaining spatial connectivity between nesting and foraging sites, and controlling the fragmentation effects of road construction on core habitats.

### 4.2 Suitable Distribution Areas and Spatiotemporal Dynamics of the Oriental Stork

Climate change significantly impacts species distribution and population dynamics (Northrup et al. 2019; Coelho et al. 2023). Based on the optimized MaxEnt model, we predicted the potential distribution of the Oriental Stork under current and future climate scenarios.

Under current conditions, suitable habitats are mainly distributed in eastern China, covering 106.57 × 10^4^ km^2^ (11.1% of China's land area), consistent with its known range. Suitable areas are concentrated in the southern Northeast Plain, North China Plain, Middle‐Lower Yangtze River Plain, Pearl River Delta, and western Taiwan Plain—regions with low terrain, well‐developed water systems, and abundant wetlands, matching the species' dependence on shallow‐water foraging habitats and isolated tree nesting sites (Yang et al. 2022). Highly suitable areas (9.49 × 10^4^ km^2^) are clustered in traditional wintering and breeding grounds, indicating core habitat coverage, though human activities may pose ongoing pressure.

Under future scenarios, total potential suitable habitat is projected to generally expand, with magnitude varying across scenarios and periods. The largest expansion occurs under SSP5‐8.5 during 2061–2080 (+8.33 × 10^4^ km^2^, 7.82%), the smallest under SSP2‐4.5 during 2041–2060 (0.22%). Highly suitable areas expand most under SSP1‐2.6_2070s (9.49 to 13.26 × 10^4^ km^2^), moderately suitable areas peak under SSP5‐8.5_2090s (35.50 × 10^4^ km^2^), and low suitability areas expand most under SSP1‐2.6_2090s (70.21 × 10^4^ km^2^).

Dynamic change analysis reveals stable areas in the North China Plain, Huang‐Huai‐Hai Plain, and Middle‐Lower Yangtze River Plain. Decreased suitability occurs in south‐central Liaoning, central Shandong, and northern Jiangsu—edge zones more sensitive to climate change—while increased suitability occurs in Heilongjiang, Jilin, eastern Inner Mongolia, and Taiwan, indicating expansion toward higher latitudes and altitudes. The habitat centroid, currently near Guzhen County, Anhui (117°44′34″ E, 33°47′42″ N), exhibits a projected northeastward displacement (16.82–77.22 km), most pronounced under SSP5‐8.5_2050s. However, whether this expansion translates into actually available habitat requires cautious evaluation in light of specific ecological processes (Radosavljevic and Anderson 2014). It is also important to note that our future projections assume land use and human disturbance factors remain at current levels, which is a conservative scenario. In reality, future land use conversion and infrastructure development may offset or even reverse the climate‐driven expansion of suitable habitat. Therefore, while our results suggest climate‐driven northeastward expansion of climatically suitable areas, the actual habitat available to the species will depend on the interplay between climate change and future land‐use dynamics.

MESS analysis shows most areas retain similarity with current conditions (MESS > 0, marginal within‐range areas > 97%), with low extrapolation uncertainty. MESS values decrease over time, dropping to 9.50 under SSP5‐8.5_2090s. Severe extrapolation (< −50) accounts for < 0.04%, mainly in southwestern Xinjiang, western Tibet, and eastern Taiwan. MOD analysis identifies precipitation seasonality (Bio15, 79.24%) and precipitation of the wettest month (Bio13, 12.35%) as primary extrapolation drivers (combined 92.55%), far exceeding topography (4.52%) and temperature (1.85%). Bio15 dominates in eastern monsoon region core zones, reflecting the critical constraint of precipitation variability on dry season hydrology, while mean diurnal temperature range (Bio2) emerges as a local dominant factor in specific scenarios (e.g., SSP1‐2.6_2090s, SSP5‐8.5_2090s).

In summary, the Oriental Stork's suitable habitat shows overall expansion, northeastward shift, and variation across scenarios. Precipitation factors (especially dry‐season precipitation) are key drivers of distribution changes and extrapolation uncertainty. Conservation implications: (1) stable areas (North China Plain, Middle‐Lower Yangtze River Plain) as core protection zones; (2) proactive spatial planning and ecological corridors in the Northeast expansion region; (3) hydrological regulation to maintain dry‐season wetland water levels; and (4) long‐term monitoring for adaptive management under climate uncertainty.

### 4.3 Priority Conservation Areas and Conservation Gaps

Land use change significantly impacts species distribution (Cardinale et al. 2012), yet many MaxEnt‐based studies focus on bioclimatic, soil, and topographical factors while neglecting land use type. For wetland species like the Oriental Stork, habitat quality is crucial—habitat loss is a primary driver of biodiversity decline (Francoso et al. 2015), and conservation efforts aim to maintain biodiversity and ensure sustainable use (Adhikari et al. 2019). This study transcended single‐species distribution models by coupling the optimized MaxEnt model with the HSI‐InVEST integrated model, achieving spatial quantification of habitat quality and accurate identification of priority conservation areas.

This integrated model combines the strengths of HSI (explicit ecological factor representation) and InVEST (spatial decay mechanism for threats). The HSI sub‐model, through weight optimization, captured the dominant role of altitude (80.3% contribution in MaxEnt), filling the gap where InVEST cannot reflect elevation effects. The InVEST sub‐model integrated spatial information on human threats (roads, construction land), accurately reflecting anthropogenic disturbance impacts. The 7:3 integration weight balanced natural factors and anthropogenic threats. Validation showed that integrated model predictions reached 0.97 in core habitats (e.g., Poyang Lake) and dropped to 0.35 in urban areas (e.g., Shanghai), aligning more closely with ecological expectations than single models, with high‐quality habitats (≥ 0.8) highly coinciding with known distribution areas.

Priority conservation areas are mainly concentrated in lake beaches and floodplain wetlands of the North China Plain, Huang‐Huai‐Hai Plain, and Jianghuai region, including: Baiyangdian wetland in south‐central Hebei; bottomlands around Nansi Lake and Dongping Lake in western Shandong; Hongze Lake, Yancheng wetlands, and Lixiahe wetland in northern Jiangsu; bottomlands along the Huaihe River and river network wetlands in the Huaibei Plain in northern Anhui. Priority areas are also distributed in the southern Songnen Plain, Liaohe Plain wetlands, northern Jianghan Plain, wetlands around Dongting Lake and Poyang Lake, the Pearl River Delta, and coastal wetlands in western Taiwan. These areas share common characteristics: low altitude, moderate distance to lakes, relatively far distance to roads, and land use dominated by natural wetlands, mudflats, and paddy fields—highly consistent with dominant factor analysis results. From a conservation biogeography perspective, these areas are important nodes in the East Asian‐Australasian Flyway, possessing high hydrological connectivity and habitat heterogeneity to meet the species' spatial separation requirements for foraging and nesting sites.

Based on these findings, we recommend implementing strict spatial control over priority conservation areas, maintaining core wetland water levels and nesting habitats through hydrological regulation and ecological restoration, constructing ecological corridors between the North China Plain and Northeast Plain to ensure migratory connectivity, and promoting a community co‐management model to achieve synergistic development of conservation and local livelihoods. Under the “30 × 30” framework, our gap analysis reveals that 132,448 km^2^ of priority conservation areas currently fall outside the existing protected area network, representing a substantial conservation gap. However, to achieve the “30 × 30” target—defined as protecting at least 30% of the total priority habitat area—the additional area that would need to be brought under protection is 27,715 km^2^ (149,618 km^2^ × 30% − 17,170 km^2^). This more refined target acknowledges that protecting all 132,448 km^2^ of unprotected priority habitat may not be immediately feasible, while 27,715 km^2^ represents a more focused and achievable conservation goal. We recommend that the highest‐quality gap areas be prioritized for inclusion in the protected area network through the establishment of new reserves and expansion of existing boundaries to meet the “30 × 30” target while ensuring the long‐term persistence of the Oriental Stork.

### 4.4 Limitations

Despite satisfactory predictive performance through MaxEnt optimization, environmental variable screening, and integrated model construction, several limitations remain: (1) MaxEnt predicts maximum potential distribution under ideal conditions rather than actually occupied ecological space, as species distribution is often constrained by factors not included in the model (e.g., interspecific competition, predation). (2) Although feature classes and regularization multiplier were optimized using the “kuenm” R package, selecting the optimal model with omission rate < 5% and smallest ΔAICc to reduce complexity and overfitting risk (Radosavljevic and Anderson 2014), sensitivity to sampling bias cannot be completely eliminated; while ENMTools corrected for spatial autocorrelation (one representative point per grid cell), distribution data from public databases and literature may contain geographical sampling bias (e.g., more records near roads and protected areas), potentially affecting accuracy in depicting environmental response curves (Fourcade et al. 2014). (3) Because the Oriental Stork is a migratory species, our occurrence records were not separated by breeding, migration, or wintering periods. Consequently, the MaxEnt model predicts an integrated distribution across the annual cycle rather than season‐specific habitats. While this approach is appropriate for identifying overall suitable areas and conservation priorities at the macro‐scale, we caution against interpreting the results as representing nesting, foraging, or stopover habitats individually. Future studies incorporating seasonal occurrence data would enable more refined, phenologically explicit conservation planning. (4) Future climate projections in this study were derived from a single GCM, BCC‐CSM2‐MR. While this model was selected for its demonstrated performance in simulating climate over China and its widespread use in regional impact studies, we acknowledge that relying on a single GCM introduces uncertainty, as different climate models can produce substantially different projections of species habitat suitability. Multi‐model ensemble (MME) approaches have been shown to provide more robust predictions by reducing GCM‐specific biases, and we recognize this as an important direction for future research. (5) Factors such as land use data timeliness, completeness of threat sources, and species occurrence point bias may affect model accuracy; the HSI model incorporated only two factors (altitude, land use), neglecting climate, hydrology, and food resources, while threat attenuation parameters in the InVEST model were based primarily on previous studies and may not fully apply to different regions of China, and the 1 km resolution may fail to capture fine‐scale features such as small wetlands. (6) Future research should incorporate more ecologically relevant factors, conduct multi‐scenario dynamic simulations, adopt multi‐scale nested approaches, and strengthen efforts in identifying ecological corridors and evaluating conservation effectiveness; subsequent studies should employ connectivity modeling methods to identify key conservation corridors and prioritize them, followed by targeted ecological restoration measures to mitigate corridor fragmentation and maintain landscape functional connectivity. Overall, the integrated MaxEnt and HSI‐InVEST model coupling effectively assessed habitat quality spatial patterns for the Oriental Stork, and the identified priority conservation areas highly coincide with known ecological requirements. The findings provide scientific references for conservation planning of the Oriental Stork and wetland waterbird protection.

## 5 Conclusions

Under different climate scenarios, MaxEnt model predictions indicate that potential suitable habitats for the Oriental Stork are projected to expand in the Northeast Plain, Songnen Plain, and eastern Inner Mongolia, although the magnitude varies across scenarios and time periods. Altitude is the most important environmental driver, while precipitation variables (precipitation of the wettest month and coldest quarter) are key factors driving future distribution changes.

By overlaying these predictions with habitat quality data from the HSI‐InVEST integrated model, we delineated priority conservation areas (“dual‐high areas”: suitability ≥ 0.4, habitat quality index ≥ 0.8). These are concentrated in lake beach and floodplain wetlands of the North China Plain, Huang‐Huai‐Hai Plain, and Jianghuai region, including Baiyangdian Lake, Nansi Lake, Hongze Lake, Yancheng Wetlands, wetlands along the Huaihe River, areas surrounding Dongting Lake and Poyang Lake, the Pearl River Delta, and coastal wetlands in western Taiwan. These areas should be designated as core zones for long‐term protection, with strict spatial control and hydrological regulation.

Furthermore, we recommend establishing an annual monitoring platform based on GIS and remote sensing to track dynamic habitat changes in priority conservation areas and potential expansion zones, assess the impacts of evolving climate scenarios on wetland hydrology and land use patterns, and provide data support for adaptive governance balancing conservation and development. Specifically, our spatial overlay analysis reveals that 132,448 km^2^ of priority conservation areas currently fall outside the existing protected area network. To meet the “30 × 30” representation target, the additional area requiring protection is 27,715 km^2^. We recommend that the highest‐quality gap areas be prioritized for inclusion in the protected area system through the establishment of new reserves and expansion of existing boundaries to meet the “30 × 30” target while ensuring the long‐term persistence of the Oriental Stork. This integrated modeling and management framework systematically couples species distribution models with habitat quality assessment models, offering robust theoretical support for optimizing in situ conservation strategies and planning ecological corridors for the Oriental Stork under future climate scenarios.

## Author Contributions

**Pengzhou Chen:** data curation (lead), investigation (lead), methodology (lead), visualization (lead), writing – original draft (lead). **Haoyu Ma:** data curation (supporting), investigation (supporting), methodology (supporting), visualization (supporting). **Yue Fang:** data curation (supporting), visualization (supporting). **Jiaxiang Chang:** data curation (supporting), visualization (supporting). **Donglai Hua:** supervision (equal), writing – review and editing (equal). **Hao Liu:** funding acquisition (lead), supervision (equal), writing – review and editing (equal).

## Funding

This study was supported by The Mianyang Normal University Doctoral Start‐up Fund Project [QD2023A33]. The project “Investigation on the Wintering Population and Habitat of the Scaly‐sided Merganser in Sichuan” [071/HX2024164].

## Conflicts of Interest

The authors declare no conflicts of interest.

## Supporting information


**Table S1:** Maximum distance over each threat affecting habitat quality and its weight in China.

**Table S2:** Habitat suitability of different land use types and relative sensitivity to threat factors in China.

## Data Availability

The data that supports the findings of this study are available in the Supporting Information of this article.
