# Supplementary material for: Climate Change Impacts on the Distribution Patterns and Habitat Suitability of the Oriental Stork ( Ciconia boyciana ) in China: Implications for the “30 × 30” Conservation Targets
Source: Ecol Evol. 2026 Sep 28;16(10):e74424. doi: 10.1002/ece3.74424 (PMC13619722; doi:10.1002/ece3.74424)
Supplement: Supplementary file 1 [file ECE3-16-e74424-s001.docx]

Table S1 Maximum distance over each threat affecting habitat quality and its weight in China.

| Name | Max_Dist/km | Weight | Spatial decay type |
| --- | --- | --- | --- |
| Paddy field | 4 | 0.4 | Linear |
| Dryland | 3 | 0.5 | Exponential |
| Urban | 8 | 1 | Exponential |
| Village | 5 | 0.6 | Exponential |
| Other construction land | 6 | 0.4 | Exponential |
| Railway | 4 | 0.4 | Linear |
| Motorway | 3 | 0.4 | Linear |
