# Supplementary material for: Climate Change Impacts on the Distribution Patterns and Habitat Suitability of the Oriental Stork ( Ciconia boyciana ) in China: Implications for the “30 × 30” Conservation Targets
Source: Ecol Evol. 2026 Sep 28;16(10):e74424. doi: 10.1002/ece3.74424 (PMC13619722; doi:10.1002/ece3.74424)
Supplement: Supplementary file 2 [file ECE3-16-e74424-s002.docx]

Table S2 Habitat suitability of different land use types and relative sensitivity to threat factors in China.

| Name | Habitat quality | Paddy field | Dryland | Urban | Village | Other construction land | Railway | Motorway |
| --- | --- | --- | --- | --- | --- | --- | --- | --- |
| Paddy field | 0.7 | 0.2 | 0 | 0.6 | 0.5 | 0.5 | 0.1 | 0.2 |
| Forest land | 0.3 | 0.6 | 0.6 | 0.8 | 0.7 | 0.75 | 0.6 | 0.8 |
| Grassland | 0.6 | 0.7 | 0.7 | 0.7 | 0.6 | 0.7 | 0.15 | 0.2 |
| River and canal | 0.8 | 0.8 | 0.65 | 0.85 | 0.7 | 0.5 | 0.4 | 0.5 |
| Lake | 0.9 | 0.3 | 0.3 | 0.75 | 0.6 | 0.6 | 0.5 | 0.55 |
| Reservoir and pond | 0.8 | 0.8 | 0.65 | 0.85 | 0.7 | 0.5 | 0.5 | 0.55 |
| Permanent glacier | 0 | 0 | 0 | 0 | 0 | 0 | 0 | 0 |
| Mudflat | 0.9 | 0.5 | 0.7 | 0.7 | 0.2 | 0.5 | 0.55 | 0.6 |
| Floodplain | 0.8 | 0.5 | 0.7 | 0.7 | 0.2 | 0.5 | 0.55 | 0.6 |
| Construction land | 0 | 0 | 0 | 0 | 0 | 0 | 0 | 0 |
| Sandy land | 0 | 0 | 0 | 0 | 0 | 0 | 0 | 0 |
| Gobi | 0 | 0 | 0 | 0 | 0 | 0 | 0 | 0 |
| Saline-alkali land | 0 | 0 | 0 | 0 | 0 | 0 | 0 | 0 |
| Marshland | 1 | 0.5 | 0.5 | 0.6 | 0.3 | 0.3 | 0.1 | 0.1 |
| Bare land | 0 | 0 | 0 | 0 | 0 | 0 | 0 | 0 |
| Bare rock | 0 | 0 | 0 | 0 | 0 | 0 | 0 | 0 |
| Other land | 0 | 0 | 0 | 0 | 0 | 0 | 0 | 0 |
